# Novel Aspects in Pattern Formation Arise from Coupling Turing Reaction–Diffusion and Chemotaxis

**DOI:** 10.1007/s11538-023-01225-5

**Published:** 2023-12-01

**Authors:** Camile Fraga Delfino Kunz, Alf Gerisch, James Glover, Denis Headon, Kevin John Painter, Franziska Matthäus

**Affiliations:** 1 Frankfurt Institute for Advanced Studies and Department of Computer Science and Mathematics, Goethe-University Frankfurt, Ruth-Moufang-Str. 1, 60438 Frankfurt, Germany; 2 Department of Mathematics, Technical University Darmstadt, Darmstadt, Germany; 3 The Roslin Institute and R(D)SVS, University of Edinburgh, Edinburgh, EH25 9RG UK; 4 Dipartimento Interateneo di Scienze, Progetto e Politiche del Territorio (DIST), Politecnico di Torino, Turin, Italy

**Keywords:** Reaction–diffusion, Chemotaxis, Morphogenesis, Biological development

## Abstract

Recent experimental studies on primary hair follicle formation and feather bud morphogenesis indicate a coupling between Turing-type diffusion driven instability and chemotactic patterning. Inspired by these findings we develop and analyse a mathematical model that couples chemotaxis to a reaction–diffusion system exhibiting diffusion–driven (Turing) instability. While both systems, reaction–diffusion systems and chemotaxis, can independently generate spatial patterns, we were interested in how the coupling impacts the stability of the system, parameter region for patterning, pattern geometry, as well as the dynamics of pattern formation. We conduct a classical linear stability analysis for different model structures, and confirm our results by numerical analysis of the system. Our results show that the coupling generally increases the robustness of the patterning process by enlarging the pattern region in the parameter space. Concerning time scale and pattern regularity, we find that an increase in the chemosensitivity can speed up the patterning process for parameters inside and outside of the Turing space, but generally reduces spatial regularity of the pattern. Interestingly, our analysis indicates that pattern formation can also occur when neither the Turing nor the chemotaxis system can independently generate pattern. On the other hand, for some parameter settings, the coupling of the two processes can extinguish the pattern formation, rather than reinforce it. These theoretical findings can be used to corroborate the biological findings on morphogenesis and guide future experimental studies. From a mathematical point of view, this work sheds a light on coupling classical pattern formation systems from the parameter space perspective.

## Introduction

The capacity of systems to self-organise has been a long source of fascination, with examples spanning microbes to landscapes. Embryogenesis is a paradigm of spatial self-organisation, during which a more or less uniform population of cells arranges and differentiates into diverse tissues. Various models for self-organisation have been proposed, with the well-known reaction–diffusion system of Turing (1952) and the chemotaxis model of Keller and Segel (1970) lying at the forefront. These share a fundamental capacity to trigger self-organisation within a uniform tissue, yet are built on different assumptions regarding the behaviour of cells and their interaction with signalling components (Painter et al. 2021).

The reaction–diffusion theory of morphogenesis postulates that an initially homogeneous tissue can pattern solely through chemical reaction and diffusion (Turing 1952). An “active” cell population is not a definite requirement: the chemical system generates the spatial pattern, providing a blueprint for cell differentiation. Distilled into a minimum of two reacting and diffusing species, it requires a short-range activator and a long-range inhibitor (Gierer and Meinhardt 1972, 1974). The activator possesses autocatalytic properties that drive the reaction, with the inhibitor the brake. Counter-intuitively, the addition of diffusion breaks the symmetry of the uniform solution: short-range activator diffusion allows autocatalysis to dominate locally while inhibition suppresses at a distance and a chemical pattern forms. The last two decades have witnessed numerous morphogenesis processes in which reaction–diffusion type systems may play a significant role in patterning, e.g. Harris et al. (2005), Nakamura et al. (2006), Michon et al. (2008), Cho et al. (2011), Sala et al. (2011), Economou et al. (2012), Raspopovic et al. (2014), Walton et al. (2016), Glover et al. (2017), Kaelin et al. (2021).

Chemotaxis models rely instead on an active cell population, in the sense that the movement dynamics of cells are crucial for generating the patterned state. Originally developed in the context of slime mold aggregation, the model of Keller and Segel (1970, 1971) minimally consists of a homogeneous cell population and its chemical chemoattractant. Cells both secrete and move up the gradient of the chemoattractant, this autoattraction rounding up a dispersed population into one or more aggregates. Chemotaxis models have been proposed to explain numerous instances of self-organisation, including developmental processes ranging from vasculogenesis to skin patterning, e.g. see Painter (2019).

These two models are often considered in isolation, yet growing evidence suggests that they can operate in tandem. Mammalian and avian skin is characterised by repetitive hair or feather elements, their placement laid out during early embryonic development when the essentially uniform embryonic skin self-organises into a periodic array of bud or placode structures. This accessible system has offered a paradigm for understanding morphogenesis, in recent years integrating both experiment and theory (e.g. see Schweisguth and Corson 2019; Painter et al. 2021). Generating the pattern involves interactions between the tightly packed epithelial cells and the mesenchymal cells beneath, with presumptive placodes identified through the expression of certain genes in the epithelium and an aggregation of mesenchymal cells below. In the case of mouse hair follicles, Glover et al. (2017) have identified an interacting set of pathways, involving fibroblast growth factors (FGFs), bone morphogenic proteins (BMPs) and wingless-related integration site (WNTs), capable of inducing patterning via a reaction–diffusion type mechanism. This chemical pattern foreshadows and subsequently directs mesenchymal cell organisation, with FGF-mediated chemotaxis leading to mesenchymal aggregation. However, the dermis also has an autonomous ability to pattern, as demonstrated by mesenchyme cells retaining the capacity to aggregate on suppression of the epithelial reaction–diffusion mechanism (Glover et al. 2017) (albeit on a slower timescale, and with less regularity). Further, it is plausible that feedback into epithelial signalling can result from mesenchymal aggregation, for example studies of avian skin showing that compression by dermal aggregation induces signalling in the epithelium (Shyer et al. 2017). Laying out the feather buds also involves a coupling between chemotaxis and activator/inhibitor-like molecular components (Painter et al. 2018; Ho et al. 2019), although in that system chemotaxis-based autoaggregation appears to be the key instigator of the periodic pattern and there is no clear evidence for an autonomous pattern generator through reaction–diffusion alone.

Motivated by these examples, the question we address in this work is as follows: how are the dynamics of pattern formation altered under a mechanism involving two semi-autonomous patterning systems? In particular, we will examine a dual patterning system in which a Turing reaction–diffusion mechanism is coupled to a chemotaxis system, designed such that pattern formation can occur through chemotaxis alone, through reaction–diffusion alone, through both as competent symmetry breaking systems, or through neither system alone. The model is not intended to describe details of the real biological system involving two skin layers and several chemical species, but in a more abstract setting, explore the interaction of the two classical patterning processes. As such, we use our study to explore a number of scenarios, such as whether activator–inhibitor systems that lie outside the pattern forming space can be pushed inside through increased chemotaxis, or whether systems in which patterning occurs in the coupled system are unable to pattern when decoupled. Previous modelling has taken steps in this direction. For example, in Painter et al. (1999) the chemical output of a reaction–diffusion system was hypothesised to direct the movements of a chemotactic pigment cell population in a model for fish pigmentation patterning. However in that case there was no reverse feedback from the cells into the reaction–diffusion system. On a similar note, a more recent study derived a system including both chemotaxis and reaction–diffusion from an underlying individual-based model, though again not considering feedback from the chemotaxis population into the reaction–diffusion network (Macfarlane et al. 2020). More directly pertinent to the present study, (at least) two models have been proposed directly with the purpose of explaining experimental observations in the context of feather morphogenesis. In Michon et al. (2008) a model based on partial differential equations (PDE) was developed whereby a chemotactic population both responded to and altered the dynamics of a reaction–diffusion network. More recently, Bailleul et al. (2019) coupled a chemotactic cell population with a reaction–diffusion network of activator–inhibitor type, with the cell population upregulating both activator and inhibitor components. Both of these studies, though, primarily focused on the application of the model to explain experimental observations, and little formal analysis of the model was undertaken.

**Fig. 1:**
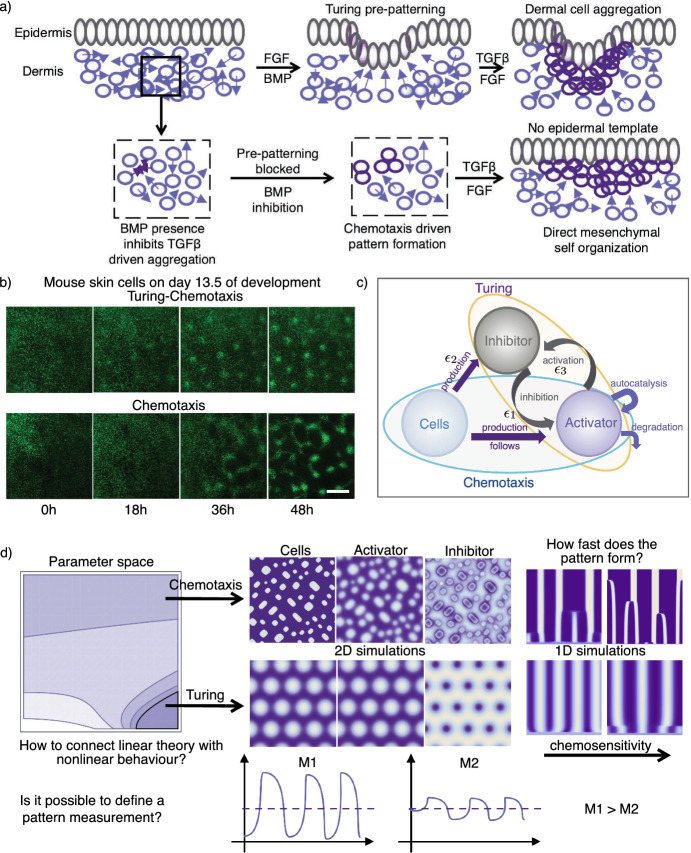
Graphical overview. **a** Epidermis and dermis interact via signalling components. Pattern formation starts with an activator (WNT/FGF) and an inhibitor (BMP), secreted by the epidermis. Dermal cells chemotax along the FGF gradient, whereby chemotaxis is mediated by TGF$\beta$. This leads to regularly distributed cell clusters at the positions where the hair follicles are formed later on. Adding FGF and inhibiting BMP prevents the Turing pre-patterning from forming, leaving chemotaxis as the only active patterning process. The cells still form patterns and the hair follicle grows, but the cell condensates are less regularly distributed and vary in diameter (Glover et al. 2017). **b** Images showing pattern formation in mouse skin after day 13.5 of development (+0, +18, +36 and +48 h). The experiments considered the full system (top row), as well as a setup with a blocked Turing system (bottom row) (Glover et al. 2017). Scale bar: 250$\upmu$ m. **c** Schematic representation of the model approach, coupling a Turing reaction–diffusion system and chemotaxis. Our simplified model considers only one cell population, one activator and one inhibitor. **d** Results of a linear stability analysis and numeric simulations: Linear stability analysis provides parameter regions for pattern formation. 2D simulations confirm the analytic results and show the variety of evolving patterns. 1D simulations show the temporal evolution of these patterns. A pattern measure *M* is defined to quantify the pattern variance (Color figure online)

In this paper we build on these studies, and in particular use a combination of linear stability and numerical analysis to obtain a deeper understanding into the implications of dual patterning mechanisms on pattern formation (Fig. 1).

The main findings of our study can be summarized as follows: (a) coupling diffusion–driven patterning and chemotaxis as proposed in our model generally enlarges the pattern region in the parameter space, and thus enhances the robustness of the patterning process, (b) an increase in chemosensitivity generally reduces the spatial regularity of the pattern in the considered simulation time, (c) on the other hand, increased chemosensitivity can speed up the patterning process for parameters inside and outside of the Turing space, (d) pattern formation in the coupled system can occur for parameter ranges where neither the Turing nor the chemotaxis system alone can independently generate pattern, (e) for some parameter settings, the coupling of the two processes can extinguish the pattern formation, rather than reinforce it.

## Mathematical Model

The formation of follicles and feathers demands interactions between two cell populations and numerous signalling molecules, that reside and operate across epithelial and mesencyhmal skin layers and potentiate pattern formation through reaction–diffusion and/or chemotaxis or other mechanical processes (Glover et al. 2017; Shyer et al. 2017; Ho et al. 2019; Bailleul et al. 2019; Painter et al. 2021). To facilitate a tractable model, we forsake a detailed model and instead distil the complexity into a more abstract formulation. Specifically, we consider a generic cell population (amalgamating cells of epithelial and mesenchymal layers) and two generic morphogens, respectively of activator and inhibitor nature (amalgamating signalling interactions). We assume cells display positive chemotaxis in response to the gradient of the activator, as well as directly modulating the signalling network through up/down-regulation of activator or inhibitor components. Further, we disregard cell proliferation, supposing the time scale of patterning is fast compared to cell proliferation; in the context of feather and hair follicle formation, the initial transformation from uniform to patterned state takes place over a relatively short time scale, i.e. the order of hours (Glover et al. 2017).

We formulate this model as a system of PDEs, where we denote the cell density by $u=u(t,x,y)$, the activator concentration by $v=v(t,x,y)$ and the inhibitor concentration by $w=w(t,x,y)$. Note that the position $(x,y)\in \Omega \subset {\mathbb {R}}^2$ assumes patterning takes place across an effectively 2D layer, and we assume time $t\in [0,T]$. The general model combines a Keller and Segel (1970) and Turing/reaction–diffusion (Turing 1952) framework, specifically

1 $$\begin{aligned} {\left\{ \begin{array}{ll} \displaystyle \frac{\partial u}{\partial t} \,&{}=\, D_{u} \nabla ^{2} u \,-\, \chi {\varvec{\nabla }} \,\cdot \,( u (1 - u/ \beta ) {\varvec{\nabla }}v)\\ \displaystyle \frac{\partial v}{\partial t} \,&{}=\, D_v \nabla ^{2} v \,+ \, f(u,v,w)\\ \displaystyle \frac{\partial w}{\partial t} \,&{}=\,D_{w} \,\nabla ^{2} w \,+\, g(u,v,w) \ . \end{array}\right. } \end{aligned}$$

For the reaction–diffusion part, $D_v$ and $D_w$ represent the diffusion coefficients of the activator and inhibitor, respectively. The functions $f=f(u,v,w)$ and $g=g(u,v,w)$ describe activator and inhibitor kinetics. Note that *f* and *g* are assumed to have a form capable of generating Turing-type instabilities (Eqs. 24–27), independently of the cell population.

For the cellular dynamics, $D_u$ is a diffusion coefficient describing random cell movement, while $\chi$ denotes the chemotactic sensitivity, i.e. a measure of the strength of the chemotactic response, with respect to the activator (as indicated by experiments where cells follow the gradient of FGF20, see Glover et al. 2017). Because there is no indication of chemotaxis towards the inhibitor in experimental studies it is omitted here. We note that the $1-u/ \beta$ factor describes a “volume-filling” form (Painter and Hillen 2002), inhibiting cells from accumulating beyond a critical cell density threshold, $\beta$; from a mathematical perspective, this limits the potential for “blow-ups”, i.e. where unrealistically concentrated cell densities emerge at the site of an aggregation.

Equation 1 require closure through appropriate initial and boundary conditions. For boundary conditions at the domain boundary, $\partial \Omega$, we adopt the standard assumption of periodic boundary conditions; for tissues such as the skin, which form a closed surface around the body, this would seem a reasonable choice. For initial conditions we choose the classic self-organising scenario of patterning from a homogeneous positive steady state, i.e. investigating the possibility of Turing instabilities. As such, we consider small randomised perturbations about a non-negative steady state. For values close to zero we enforce non-negativity in all simulations. Denoting the steady state as $(u^{*},v^{*},w^{*})$, we therefore assume initial conditions of the form

2 $$\begin{aligned} u_{0} = u^{*}+\delta _{1} \, , \quad v_{0} =v^{*}+\delta _{2} \, , \quad w_{0} = w^{*}+\delta _{3} \ . \end{aligned}$$

where $0<u_0<\beta$, as $\beta$ controls the limit of the volume filling term. The $\delta _i$’s denote uniformly distributed random numbers in the interval $-10^{-2}\le \delta _i \le 10^{-2}$, e.g., with zero mean and variance $\frac{10^{-4}}{3}$. Note that the zero mean of $\delta _1$ implies that the perturbed initial condition $u_0$ has the same mass as the cell population steady state $u^*$, in line with the mass conservation property of the equation for the cells; we also ensure this condition in our numerical simulations.

### Case Study Kinetics—The Schnakenberg-System

As a specific case study we consider reaction terms based on Schnakenberg-type kinetics (Schnakenberg 1979), albeit modified to allow cellular feedback through cellular stimulation of the activator and/or inhibitor. Following a non-dimensionalisation, see “Appendix B”, the system we consider is given by

3 $$\begin{aligned} {\left\{ \begin{array}{ll} \displaystyle \frac{\partial u}{\partial t} \,&{}=\, D_{u} \nabla ^{2} u \,-\, {\varvec{\nabla }} \,\cdot \,( \chi u (1 - u/ \beta ){\varvec{\nabla }}v)\\ \displaystyle \frac{\partial v}{\partial t} \,&{}=\, \nabla ^{2} v \,+\, \gamma ( a(1+\epsilon _{1}u)-v+\epsilon _{3}v^{2}w) \\ \displaystyle \frac{\partial w}{\partial t} \,&{}=\,D_{w} \,\nabla ^{2} w \,+\, \gamma (c(1+\epsilon _{2}u)-\epsilon _{3}v^{2}w) \ . \end{array}\right. } \end{aligned}$$

The parameter $\gamma$ denotes a scaling parameter, such that changing $\gamma$ allows activator and inhibitor reactions to operate on a distinct time scale to that of movement processes (Murray 2003). Observe that in “Appendix B” the nondimensionalised diffusion coefficients are $D_{u}^*=D_{u}/D_{v}$, $D_{w}^*=D_{w}/D_{v}$. In the case study kinetics we drop the asterisks for all parameters in Eq. 36 for simplicity of notation.

The nondimensionalisation as given in “Appendix B” is suitable for our purposes, but is certainly not unique. Our guiding principle has been to eliminate as many parameters of the model as possible in order to simplify the subsequent analysis. In particular, we have scaled the cell density by the spatially homogeneous steady state density $u^*$, which results in a fixed homogeneous steady state density (equal to one) in the nondimensional system. However, as we do not have cell proliferation, any value of $u^*$ is permissable and the nondimensionalisation removes that freedom, limiting study into the effect of the initial cell mass on the pattern performing potential of the coupled system. This might be of interest for future work, given the importance of the initial cell mass for, say, pattern formation in classical Patlak-Keller-Segel systems (e.g. see Horstmann 2003). Alternative nondimensionalisations, for instance, could use the maximum cell density $u_{\max }=\beta$ as a scaling value for the cell density.

We note that in the case $\epsilon _1 = \epsilon _2 = 0$ and $\epsilon _3 = 1$, the reaction–diffusion component of Eq. 3 forms a classical Turing system of Schnakenberg-type with background production of the chemicals through *a* and *c*, and pattern formation is possible under a relatively simple set of conditions for $a, c, D_w$. Coupling the system through either $\epsilon _1 > 0$ or $\epsilon _2 >0$ assumes that the cell population regulates the signalling system through directly or indirectly upregulating activator or inhibitor. The parameter $\epsilon _{3}$ is instead used to control the autocatalytic interactions between activator and inhibitor, critical for Turing-type patterning. In particular, we note that by setting $\epsilon _3 = 0$, the full system becomes unable to pattern through the reaction–diffusion sub-system. However, if $\epsilon _1>0$ then patterning remains possible through a classical chemotaxis-driven mechanism, whereby the cell population produces its own attractant.

The PDE system Eq. 1, both generally and under the specific kinetics given in Eq. 3, will be analysed via standard linear stability analysis in the next sections. We subsequently study the system behaviour numerically, exploring longer time scale dynamics once the system has evolved beyond the region of validity for the linear stability analysis.

## Results

### Linear Stability Analysis for the Full Model

In the following we conduct a linear stability analysis to predict the scenarios under which Eq. 1 can lead to pattern formation. Before starting, we note a number of conveniences that have been adopted, primarily to simplify presentation of the analysis. First, we restrict to a one-dimensional domain which is implicitly assumed to be large with respect to the characteristic scale of any potential spatial patterns (in effect, “infinite”). This minimises any influence from the boundary conditions or domain size on patterning, and is also reasonable in the context of follicles and feathers, where structures emerge with a separation of about 200 microns, but the tissue itself spans millimetres. Second, we presume $\beta$ is large (i.e. $\beta \longrightarrow \infty$), equivalent to assuming that the cell density remains far below the packing density. We note that it is relatively straightforward to relax these simplifications, but they do not substantially alter the nature of the conclusions stated below.

First, we lay down some terminology to describe parameter regimes in which standard linear stability analysis (e.g. Murray 2003) indicates possible pattern formation. As noted, the model 1 builds on two classic pattern formation subsystems. In the absence of the cell population ($u=0$) we reduce to a standard two-variable reaction–diffusion model of Turing type (Turing 1952), see Eq. 23; classic textbook analysis yields a well-known set of conditions for the so-called diffusion driven instability and we refer to the corresponding parameter space under which this occurs for this two variable model simply as the *Turing space*, see “Appendix A”. In the absence of reactions between the two molecular species ($f(u,v,w) = f(u,v), g(u,v,w) = g(u,w)$), the system reduces to a simple chemotaxis model of Keller–Segel type (Keller and Segel 1970), see Eq. 29; pattern formation can arise through a chemotaxis-driven instability, and we will refer to the corresponding parameter space for this two variable model simply as the *chemotaxis space*, see “Appendix A”. System 1 nontrivially combines these two classic models. The conditions for instability replace those for each of the submodels 23 and 29, although we note that they will coincide in specific cases. The corresponding parameter space for patterning in 1, which we refer to as the *combined model space*, cannot be trivially deduced from the Turing or chemotaxis space: conceivably, a set of parameters defining molecular reaction rates that lie outside (inside) the Turing space of the two species Turing model could potentially lie inside (outside) the combined space when combined with the cell population and chemotaxis mechanisms.

For Eq. 1 under general functions *f* and *g*, we assume (at least one) positive homogeneous steady state solution $(u^{*},v^{*},w^{*})$, where conservation of mass determines $u^*$ from the initial cell distribution as above, and $v^{*}$ and $w^{*}$ satisfy

4 $$\begin{aligned} f(u^{*},v^{*},w^{*})\,&=\,0 \end{aligned}$$

5 $$\begin{aligned} g(u^{*},v^{*},w^{*})\,&=\,0 \ . \end{aligned}$$

We perform a standard Turing type linear stability analysis, as described above. Letting ${\bar{U}}=({\bar{u}},{\bar{v}},{\bar{w}})$ denote the small perturbations of the steady state, linearising and looking for solutions of the form ${\bar{U}} \sim e^{\lambda t} e^{ikx}$, leads to the eigenvalue problem $\det {(S-\lambda I)}=0$, where the stability matrix *S* is given by:

6 $$\begin{aligned} S = \begin{bmatrix} -D_{u}k^{2} &{} \chi u^* k^{2} &{} 0\\ f_{u} &{} -D_{v} k^{2} + f_{v} &{} f_{w}\\ g_{u} &{} g_{v} &{} -D_{w}k^{2}+ g_{w} \end{bmatrix} \, , \quad \quad \quad k\in {\mathbb {R}}^{+}\ . \end{aligned}$$

Conditions for the steady state to be stable under a homogeneous perturbation correspond to conditions for the eigenvalues to have negative real parts when $k=0$. It is easily shown that this leads to the same first two conditions in the Turing space above, i.e. conditions in Eqs. 24 and 25. For instability following an inhomogeneous perturbation, we return to the stability matrix Eq. 6 and consider the eigenvalue problem $\det {(S-\lambda I)}=0$ for wavenumbers $k>0$. We require at least one root of the characteristic polynomial $p(\lambda )$ to have a positive real component for at least one value of *k*, where

7 $$\begin{aligned} p(\lambda )=\lambda ^{3} + A\lambda ^{2} + B\lambda + C =0 \, , \end{aligned}$$

and *A*, *B*, and *C* are coefficient functions of the wave numbers $k^{2}$ (see “Appendix C”). Given that we require at least one eigenvalue with real positive part, one of the Routh–Hurwitz conditions should be broken, which in this case can be $C<0$ or $AB-C<0$, due to positivity of *A*. We note that here that we consider instabilities both of stationary pattern type, i.e. eigenvalues have positive real parts and negligible imaginary components, and of Turing-wave type, with nonneglible imaginary components. In the latter case, emerging patterns may initially oscillate in time but still form a spatially periodic pattern and can therefore represent a plausible path towards laying out a pattern. These two conditions are studied in the following two subsections.

#### The Condition C<0

Considering the case $C<0$ we find two possible routes to pattern formation (for details see “Appendix C”). In particular, patterns can arise either in the case

8 $$\begin{aligned} \chi u^* (g_u f_w - f_u g_w) > D_u (f_v g_w - f_w g_v). \end{aligned}$$

where the right hand side is positive from Eq. 25. Alternatively, patterns can arise when the following coupled pair of conditions is satisfied:

9 $$\begin{aligned}{} & {} \chi u^* D_w f_u + D_u (D_w f_v +D_v g_w) > 0 \end{aligned}$$

10 $$\begin{aligned}{} & {} \left( \chi u^* D_w f_u + D_u (D_w f_v +D_v g_w)\right) ^2 > 4 D_u D_v D_w \nonumber \\{} & {} \quad \times \left[ \chi u^* (f_u g_w-f_w g_u) + D_u \left( f_v g_w -f_w g_v \right) \right] \end{aligned}$$

Let us first consider these conditions in light of the results from the classic models (“Appendix A”). Suppose $\chi = 0$ (no chemotaxis). Clearly, there exists no route to pattern formation through Eq. 8, while Eqs. 9–10 simply reduce to the Turing space instability conditions, i.e. Eqs. 26–27. This is logical, since any boost from chemotaxis is eliminated and patterning must be driven through activator/inhibitor interactions alone. The same scenario occurs under $f(u,v,w)=f(v,w)$ and $g(u,v,w)=g(v,w)$, i.e. where there is no cellular regulation of the signalling network. This effectively decouples the chemotactic population from the signalling system and, again, pattern formation must be driven through activator/inhibitor interactions alone. Finally, consider $g(u,v,w)=0$, i.e. no kinetics for the inhibitor component. Here, we reduce down to the single condition Eq. 31, i.e. the condition for instability for the chemotaxis space of chemotaxis-driven autoaggregation. This is again as we would intuitively expect, since the critical activator–inhibitor interactions have been eliminated and chemotaxis can only be driven through chemotaxis-driven aggregation.

#### The Condition AB-C<0

Our approach here is very similar to the one in Othmer and Scriven (1969). Denoting $q=k^2$, we write $AB-C$ as a third degree polynomial *R*(*q*),

11 $$\begin{aligned} R(q)=a_{2}q^{3}+b_{2}q^{2}+c_{2}q+d_{2} \end{aligned}$$

with turning points ${\bar{q}}_{1,2}$ and the inflection point ${\bar{q}}_{3}$. As shown in “Appendix C”, $R(q)<0$ for at least one *q*, if its coefficients satisfy:

12 $$\begin{aligned}&b_{2}<0 \quad \text {and} \quad R({\bar{q}}_{1})<0 \, , \quad \text {or}\nonumber \\&c_{2}<0 \quad \text {and} \quad b_{2}>0 \quad \text {and} \quad R({\bar{q}}_{3})<0\, . \end{aligned}$$

Stating these in an algebraic form as in Eqs. 8–10 is of limited usefulness. Rather, we remark on some special configurations. We are looking for the case in which at least one real positive root is possible (Table 3). First, note that $b_{2}$ depends on the sign of the functions $f_{u}$, $f_{v}$ and $g_{w}$, and when all of them are negative, $b_{2}$ will be positive. The case $c_{2}>0$ happens for $D_{w}f_{v}+D_{v}g_{w}<0$ and $f_{u}f_{v}+f_{w}g_{u}>0$. Taking $D_{w}=D_{u}=1$ and $f_{u}=0$, it is only possible to have $b_{2}>0$. For $b_{2}>0$, reaction–diffusion instability is possible only if $c_{2}<0$ is satisfied. However, in the same special case for $c_{2}<0$, if $f_{w}g_{u}<0$, there is a critical chemosensitivity given by

13 $$\begin{aligned} \chi >\chi _c=2 \frac{ (f_{v}+g_{w})^2+(f_{v}g_{w}-f_{w}g_{v})}{f_{w}g_{u}} \ , \end{aligned}$$

while for $g_{u}=0$ there is no critical chemosensitivity.

### Some Insights from the Linear Stability Analysis of the Full Model

The range of scenarios under which pattern formation becomes possible for more general interactions clearly becomes more complex, and we will primarily resort to a specific case study below. However, it is possible to make some general remarks as follows:

#### Pattern Formation is Possible When $D_v=D_w$, or Even $D_w =0$

The coupling of the system to chemotaxis raises the possibility that pattern formation can occur when signalling components have equal diffusion coefficients, a complicating requirement in classical activator–inhibitor systems. These observations most straightforwardly follow from Eq. 8, which provide a route to pattern formation independent of $D_w$ and essentially defining a level of chemotaxis for which pattern formation will arise. In the light of this, Eq. 8 can be viewed somewhat analogously to the simple chemotactic instability condition Eq. 31: the chemoattractant production ($f_u$ in Eq. 31) is now replaced by a term that amalgamates the cellular regulation of both activator and inhibitor components, while the chemoattractant decay ($f_v$ in Eq. 31) is now a “system-level decay” based on the activator–inhibitor signalling interactions. We stress, though, that Eq. 8 does not necessarily represent a minimum on the chemotaxis strength for for pattern formation to occur. As highlighted above, for $\chi =0$ patterning remains possible through Eqs. 9 and 10, if signalling interactions lie within the Turing space. Indeed, we could even have pattern formation for $\chi < 0$.

#### The Strict Requirements on Activator–Inhibitor Interactions for Patterning can be Relaxed

Adding chemotaxis also relaxes the strict requirements placed on activator–inhibitor interactions, i.e. the specific sign structures Eq. 28 and the necessity of autocatalyis of the activator. In other words, an activator could simply correspond to a network component that upregulates the inhibitor, without self-activating properties ($f_v >0$), provided the chemotactic sensitivity is sufficiently strong to satisfy either Eq. 8 or the pair conditions Eqs. 9 and 10.

#### The Addition of Chemotaxis can Suppress Diffusion–Driven Pattern Formation

To observe this, consider the case where $f_u=0$, $g_u \ne 0$ (no cellular regulation of the activator, but regulation of the inhibitor) and parameters lying in the Turing space (Eq. 26, 27). The Eq. 8 is not satisfied, while Eqs. 9 and 10 reduce to the single condition

$$\begin{aligned} D_u \left( D_w f_v +D_v g_w\right) ^2 > 4 D_v D_w \left[ -\chi u^* f_w g_u + D_u \left( f_v g_w -f_w g_v \right) \right] \end{aligned}$$

Lying in the Turing space, the above is satisfied when $\chi = 0$. However, for $f_w g_u >0$ it is clear that a threshold $\chi >0$ can be found that excludes the above. Of course, we should note that this does not guarantee no patterning in this case (as we have not yet considered the polynomial arising from the Routh-Hurwitz condition, see Eq. 37), however it does demonstrate the capacity of chemotaxis to suppress known routes to pattern formation.

### Linear Stability Analysis for the Chemotaxis-Schnakenberg System

The previous section supposed general functions *f* and *g* and, while some insight is possible, deeper analysis and understanding is hindered by the array of potential sign combinations in the various derivatives (i.e. $f_{u}$, etc.). To allow for a more focused analysis we turn to a case study that restricts the form in the kinetic interactions. Specifically, chemical reaction kinetics are modelled using the analytically convenient Schnakenberg system (Schnakenberg 1979; Gray and Scott 1983), adapted to include cellular upregulation of the activator and/or inhibitor components, i.e. $f_{u} \ge 0$ and $g_{u} \ge 0$. This upregulation could either occur directly (e.g. through cells secreting signalling components) or indirectly (e.g. compression from cell aggregation inducing upregulation of signalling components Shyer et al. 2017). It is also possible, of course, that cells could downregulate signalling components, but we do not consider this at present. Following nondimensionalisation, the system is as presented in Eq. 3.

To investigate the distinction between Turing-driven or chemotaxis-driven pattern formation, two parameter regimes are considered with either $f_{v}$ positive or negative. As noted in “Appendix A”, two species activator–inhibitor systems require self-upregulation of the activator, i.e. $f_v>0$. Parameters yielding $f_v<0$ will therefore preclude pattern formation driven through the activator–inhibitor system alone, but the possibility remains for pattern formation through the chemotaxis boost. As a second point of focus we consider $D_{w}=1$, which stipulates activator and inhibitor have equal diffusion coefficients. The diffusion coefficients of freely diffusing molecules of similar size will have similar magnitude (Pearson 1993), making the case of equivalent molecular diffusion coefficients of practical relevance. For convenience we also generally set $D_u = 1$ and $\epsilon _{3}=1$, except where stated otherwise.

Straightforward calculation yields a single steady state $(u^*,v^*,w^*)$, given by

14 $$\begin{aligned} (u^*,v^*,w^*) \,=\, \Big (\,1\,, \, a(1+\epsilon _{1})+c(1+\epsilon _{2}) \, , \, \dfrac{c(1+\epsilon _{2})}{\epsilon _{3}[a(1+\epsilon _{1})+c(1+\epsilon _{2})]^{2}} \,\Big ) \end{aligned}$$

where we note that $u^* = 1$ follows from the initial conditions which, via the nondimensionalisation, are scaled to unity at the mean value. Note that biologically plausible parameters (i.e. generating a positive steady state) demand $a>0$, $c>0$, $\epsilon _{1}\ge 0$, $\epsilon _{2}\ge 0$, and $\epsilon _{3}>0$.

Evaluating $f_u, f_v\ldots$, we find

15 $$\begin{aligned} f_{u} \,&=\, a \epsilon _{1} \,\ge \,0 \end{aligned}$$

16 $$\begin{aligned} f_{v} \,&=\, -1 + \frac{2c(1+\epsilon _{2})}{a(1+\epsilon _{1})+c(1+\epsilon _{2})} \end{aligned}$$

17 $$\begin{aligned} f_{w} \,&=\, \epsilon _{3}[a(1+\epsilon _{1})+c(1+\epsilon _{2})]^{2} \,>\,0 \end{aligned}$$

18 $$\begin{aligned} g_{u} \,&=\, c \epsilon _{2} \,\ge \, 0 \end{aligned}$$

19 $$\begin{aligned} g_{v} \,&=\, -\, \frac{2c(1+\epsilon _{2})}{a(1+\epsilon _{1})+c(1+\epsilon _{2})} \,<\,0 \end{aligned}$$

20 $$\begin{aligned} g_{w} \,&=\, -\epsilon _{3}[a(1+\epsilon _{1})+c(1+\epsilon _{2})]^{2} \,<\,0 \, . \end{aligned}$$

Observe that under biologically feasible parameters, signs of all the above derivatives are determined with the exception of $f_{v}$ (Eq. 16), which can be either positive or negative. In the case that the activator–inhibitor system decouples from the chemotaxis system $(\epsilon _1=\epsilon _2=0)$, this simply requires $a>c$: given this, the sign structure of the submatrix formed from the *v* and *w* components is of the form of a cross activator–inhibitor system. For $a<c$, the autocatalytic process is not sufficiently powerful to allow for Turing pattern formation through the activator inhibitor system. The eigenvalues of the Jacobian matrix have a negative real part for $\epsilon _{3}$ sufficiently large. While when $\epsilon _{3}=1$ the steady-state is always stable, decreasing $\epsilon _{3}$ can lead to a neutral centre or even an unstable steady state; that is, in the diffusion-advection-free scenario the Jacobian matrix exhibits complex eigenvalues with a positive real part.

Exploiting the known sign structure for the terms forming the Jacobian matrix, we apply the linear stability analysis to the Schnakenberg model with secretion. Notably, the potential pattern forming route through $AB-C<0$ (Eq. 12) appears to be less significant: for example, letting $f_{u}=0$ and taking $D_{w}$ and $D_{u}$ at unity, we certainly have both $b_{2}>0$ (Eq. 46), and $c_{2}>0$ (Eq. 47), since here $f_{w}g_{u}>0$. Even when $f_{u}>0$, overcoming all other positive terms would require very large $f_{u}>0$ or $\chi$. As such, within the range of analysed parameters the condition $C<0$ (Eq. 43) appears to be sufficient.

### Insights from the Analysis of the Chemotaxis-Schnakenberg-System

#### The Coupled System Exhibits Pattern Conditions Beyond Classical Turing ($D_{w}>1$) and Classical Chemotaxis ($f_{u}\ne 0$) Conditions

For the Schnakenberg system with secretion, $f_{w}g_{u} \ge 0$ and we can always find a critical positive chemosensitivity when at least one of the coupling parameters $\epsilon _{1}$ or $\epsilon _{2}$ is non-zero. Suppose further that $\epsilon _{1}=0$, i.e. there is no cellular upregulation of the activator ($f_u = 0$). This places the system outside the chemotaxis space (Eqs. 30, 31, where autoaggregation requires upregulation of the attractant by the chemotactic population). Outside the Turing space, Eqs. 9 and 10 are not satisfied, yet a critical chemosensitivity can still be deduced from Eq. 8. For this system, this reduces to the simple requirement

$$\begin{aligned} \chi >\chi _{c}=D_{u}/(c\epsilon _{2}). \end{aligned}$$

Thus, patterning is possible in the coupled system outside the individual patterning regimes of the two uncoupled processes. The above condition indicates that increasing $\epsilon _2$, hence, increasing the amount of inhibitor, can induce patterning, somewhat counterintuitive on first sight. This arises through the cross-activator–inhibitor nature of the signalling interactions, a system of substrate-depletion type in which the inhibitor *w* provides the fuel for activator production. Thus, upregulation of inhibitor by the cells allows more activator to be created, in turn leading to cell aggregation.

**Fig. 2:**
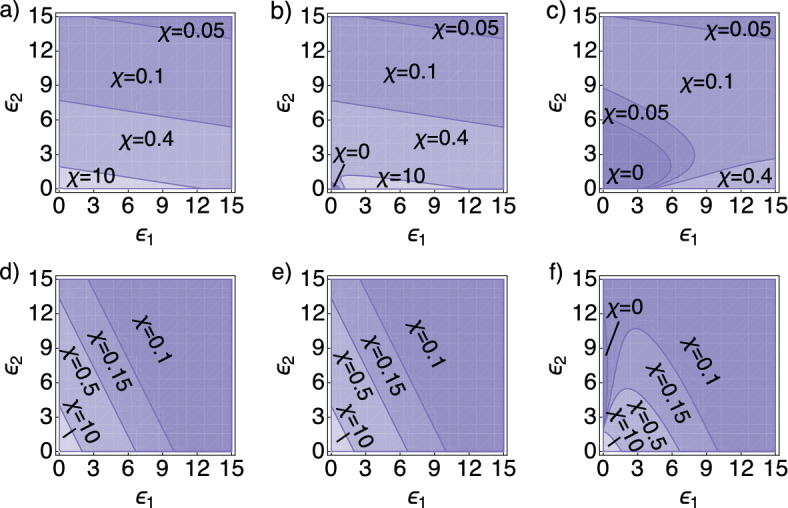
Parameter space for pattern formation for the Schnakenberg system. **a**–**f** Parameter space $\epsilon _{1} \times \epsilon _{2}$ for different choices of $\chi$ and three values of $D_{w}$: $D_{w}=1$ (left), $D_{w}=40$ (middle), and $D_{w}=600$ (right). Darker shades of purple correspond to smaller $\chi$-values. In **a**, for $\chi =0$, pattern formation does not occur, for $\chi =10$ patterning occurs for all combinations of $\epsilon _1$ and $\epsilon _2$. In **c**, $\chi =10$ is not shown, since already for $\chi =0.4$ patterning occurs for all combinations of $\epsilon _1$ and $\epsilon _2$. **a**–**c** We fixed $a=0.2$ and $c=1.3$ ($f_{v}>0$), and chose $\chi \in \{ 0, 0.05, 0.1, 0.4, 10 \}$. Note that these figures were generated with parameters for which $f_{v}>0$, i.e., a system inside the Turing space. **d**–**f** We fixed $a=1.0$ and $c=0.5$, and chose $\chi \in \{ 0, 0.1, 0.15, 0.5, 10 \}$. Note that these figures were generated with parameters for which $f_{v}<0$, i.e., a system outside the Turing space. For the cases **d** and **e** patterning is not possible for $\chi =0$ (Color figure online)

As previously mentioned, for the coupled system pattern formation is possible both in regimes $f_v <0$ and $f_v>0$, the latter a strict requirement in the activator–inhibitor system on its own. In Fig. 2 we demonstrate parameter spaces for these two regimes. In Fig. 2a–c, we consider a regime $f_v>0$, showing parameter spaces for the coupling parameters ($\epsilon _{1}$,$\epsilon _{2}$) under different choices of $D_{w}$ and $\chi$. Notably, broad regions of the parameter space show patterning under $D_{w}=1$ (Fig. 2a), with the size of this space increasing as the chemotactic sensitivity $\chi$ strengthens. Larger values of $D_w$ ($D_{w}=40$, Fig. 2b, or $D_{w}=600$, Fig. 2c) move the system towards the Turing space. Please note that in all figures the parameters $\gamma = 2200$, $\beta =4$, $D_{u}=1$, and $\epsilon _{3}=1$ are fixed unless stated otherwise. For a general overview of parameters used for every figure also see “Appendix E”.

#### $D_{w}$ Affects Patterning Only Within the Turing Space

For the system considered here, through Eq. 8 we can generate pattern formation for

$$\begin{aligned} \chi >\chi _{c}=D_{u}/(a\epsilon _{1}+c\epsilon _{2}), \end{aligned}$$

which, in essence, links chemotaxis-driven patterning to a threshold level of the combined upregulation of both the activator and the inhibitor. This follows on from above, where through the substrate-depletion system, upregulation of both activator and inhibitor components acts to fuel autocatalysis of the activator, in turn driving chemotactic aggregation. The capacity of this chemotaxis-driven condition to induce patterning is independent of inhibitor diffusion (Fig. 2). On the other hand, for high inhibitor diffusion ($D_{w}={600}$, Fig. 2f), pattern formation occurs through a combination of chemotaxis and Turing-driven instability. Through the contribution of Turing-driven instability the critical chemosensitivity drops to $\chi =0$ if the inhibitor secretion rate $3<\epsilon _{2}<12$, and the activator’s production rate $\epsilon _{1}=0$ (Fig. 2f).

#### Increasingly Influential Activator–Inhibitor Dynamics can Extinguish Chemotaxis-Driven Patterning

The parameter $\epsilon _{3}$ was introduced as a means of controlling the critical autocatalysis within the activator–inhibitor system: in the extreme scenario of $\epsilon _3 =0$, inhibitor dynamics decouples from those for cells and activator, so that the model essentially reduces to a classical autoaggregation model, with pattern formation possible only via condition in Eq. 31. Increasing $\epsilon _{3}$ increases the coupling between activator and inhibitor and, within certain parameter regimes, this can reduce the patterning space. An example of this is provided in Fig. 4a where, for example, a parameter combination $(\epsilon _1,\epsilon _2,\chi )$ that generates pattern formation for negligible $\epsilon _3$ is pushed outside the pattern forming space for larger $\epsilon _3$.

**Fig. 3:**
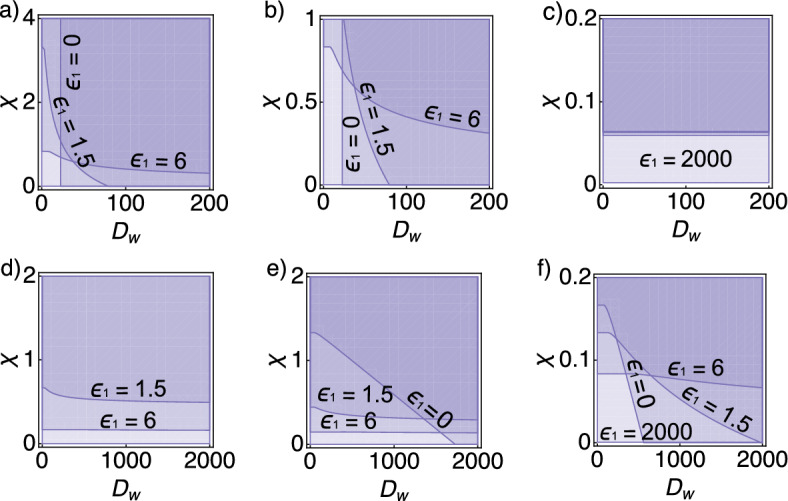
Parameter space for pattern formation for the Schnakenberg system. **a**–**f** Parameter space $D_{w} \times \chi$ and three values of $\epsilon _{2}$: $\epsilon _{2}=0$ (left), $\epsilon _{2}=1.5$ (middle), and $\epsilon _{2}=12$ (right). In all panels the parameter $\epsilon _{1}$ assumes the values $\epsilon _{1} \in \{0,\, 1.5, \, 6, \,2000 \}$, whereby the pattern region for $\epsilon _{1}=2000$ corresponds in all cases to the entire domain and is indicated by the lightest shade of purple. In **c**, all smaller values of $\epsilon _{1}$ result in a very similar pattern region, only the very large value of $\epsilon _{1}=2000$ yields patterning in entire $D_w\times \chi$ space. **a**–**c** We fixed $a=0.2$ and $c=1.3$ ($f_{v}>0$), and varied $\epsilon _{1}$ in $\{ 0, 1.5, 6, 2000 \}$. Note that the axis $D_{w}$ lies on the interval 0 to 200 for all plots, while the $\chi$ axis interval is different for each panel; **d**–**f** We fixed $a=1.0$ and $c=0.5$ ($f_{v}<0$), and varied $\epsilon _{1}$, e.g., $\epsilon _{1}\in \{ 0, 1.5, 6, 2000 \}$. Note that the axis $D_{w}$ lies on the interval 0 to 2000 for all plots, while the $\chi$ axis interval is different for each panel (Color figure online)

Generally, increasing cell-signalling coupling parameters increases the pattern-formation space. As shown in the previous section, coupling chemotaxis to a reaction–diffusion system increases the number of routes to pattern formation and, intuitively, generally acts to increase the pattern space. In the following we explore the $\chi \times D_w$ parameter space under regimes $f_v>0$ (Fig. 3a–c), and $f_v<0$ (Fig. 3d–f) as the degree of coupling is changed.

#### System Parameters Distinctly Affect the Parameter Space Inside and Outside the Turing Space

For given $\epsilon _{1}>0$ and $\epsilon _{2}=0$, we have a dependency on both $D_{w}$ and $\chi$. Hereby, we observe a transition point around $\chi \approx 1$ and $D_{w}\approx 20$: for $\chi$ and $D_{w}$ below the transition point, increasing $\epsilon _{2}$ decreases the pattern region, while for $\chi$ and $D_{w}$ above the transition point increasing $\epsilon _{2}$ increases the pattern region (Fig. 3a). For increasing $\epsilon _{2}$ we still observe a transition point, but the effect of $\chi$ and $D_{w}$ on the pattern region for different $\epsilon _{1}$ is smaller. The cases $\epsilon _{2}=0.5$ and $\epsilon _{2}=1$ exhibit a chemosensitivity-dependent region; increasing the inhibitor’s production rate decreases the critical chemosensitivity, but increases the critical $D_{w}$ (Fig. 3b, c). When the parameters *a* and *c* are set such that the system is outside the Turing space, the pattern region depends mostly on the chemosensitivity, and patterns appear only for at least one of the coupling parameters $\epsilon _{1}\ne 0$ or $\epsilon _{2}\ne 0$. Also, the critical chemosensitivity for fixed $\epsilon _{1}=0$ is smaller for $\epsilon _{2}=1$. This makes sense, because for parameters in which $f_{v}<0$, the condition $c_{1}<0$ in this parameter case ($\epsilon _{1}=0$, $c=0.5$, and $a=1$) is described in terms of a critical chemosensitivity $\chi >\chi _c=1/(0.5\epsilon _{2})$, and therefore increasing $\epsilon _{2}$ decreases $\chi _{c}$ (Fig. 3d, f). Finally, taking both coupling parameters $\epsilon _{1,2}$ equal to zero results in the diffusion coefficient $D_{w}$ being the only parameter that affects the pattern region.

#### Increasing the Activator–Inhibitor Reaction Parameter $\epsilon _{3}$ can Decrease the Pattern-Formation Space

**Fig. 4:**
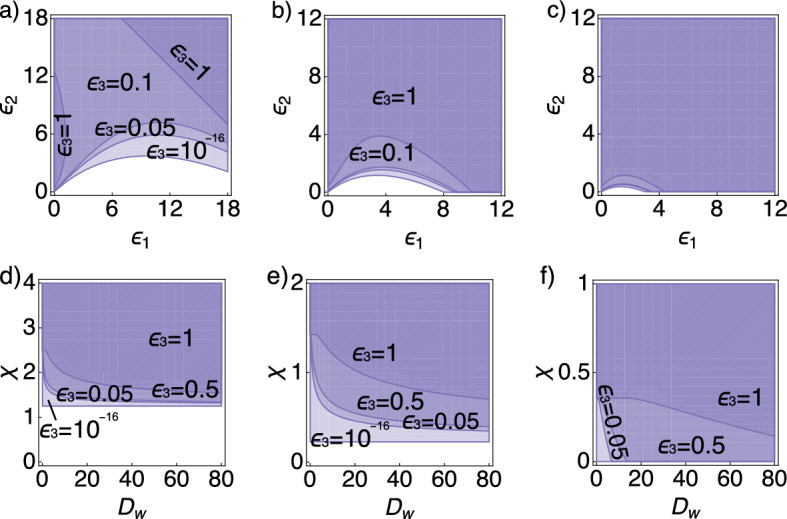
Parameter space for pattern formation for the Schnakenberg system with fixed $a=c=0.2$. In all panels the parameter $\epsilon _{3}$ assumes the values $\epsilon _{3} \in \{ 10^{-16}, \, 0.05, \, 0.5, \, 1 \}$, whereby the pattern region for $\epsilon _{3}=10^{-16}$ corresponds in all cases to the region indicated by the lightest shade of purple. **a**–**c** The contour lines indicate $\epsilon _{3}$ for varying $\epsilon _{1} \times \epsilon _{2}$ with fixed $D_{w}=1$ and considering different choices of $\chi$: $\chi =0.2$ (left), $\chi =0.5$ (center), and $\chi =1$ (right). In **b**, **c**, all smaller values of $\epsilon _{3}$ result in a very similar pattern region. Note the different choice of plotting range for each panel. **d**–**f** The contour lines indicate $\epsilon _{3}$ for varying $D_{w} \times \chi$ with fixed $\epsilon _{1}=1.5$ and considering different values of $\epsilon _{2}$: $\epsilon _{2}=0$ (left), $\epsilon _{2}=1.5$ (center), and $\epsilon _{2}=12$ (right). Note the different choice of $\chi$ plotting range for each panel (Color figure online)

For small chemosensitivities ($\chi =0.2$) we observe that increasing $\epsilon _{3}$ can decrease the pattern-formation space (Fig. 4a). For larger chemosensitivities ($\chi =0.5$ or $\chi =1$) the same effect can be observed, but to a lesser extent (Fig. 4b, c). Interestingly, for small $\chi$ ($\chi =0.2$) and small $\epsilon _{2}$ pattern formation occurs only for small or large values of $\epsilon _1$, but is absent for an intermediate range of $\epsilon _1$ (Fig. 4a). In addition, even though we are outside the Turing space, pattern formation depends on the secretion parameters $\epsilon _{1,2}$, as well as on $D_{w}$, which reflects, in the nondimensionalised system, the relation between the diffusion coefficients of the activator and inhibitor (Fig. 4e, f). The reason is that all three parameters affect the gradient of the activator, which is used by the cells as a chemotaxis cue.

### Simulations

Numerical simulations are performed as a means of both testing the results from the linear stability analysis and exploring long-term dynamics. The numerical method is described in “Appendix D”, and we focus on the nondimensional secretion-chemotaxis Schnakenberg system (Eq. 3) where the parameters $\gamma = 2200$, $\beta =4$, $D_{u}=1$, and $\epsilon _{3}=1$ are fixed for all simulations, unless stated otherwise. Initial conditions consist of a random perturbation (Eq. 2) around the steady-states (Eq. 14) and we simulate the system up to a maximum non-dimensional time $T=0.5$ (2D) or $T=0.4$ (1D). For how this translates to dimensional time, under plausible parameter choices, please refer to the discussion.

Furthermore, we define a heterogeneity measurement *M* (Eq. 21), quantifying the average distance of the cell density from the mean density (with similar measurements, not stated, for the chemical concentrations). Specifically,

21 $$\begin{aligned} M(t) = \frac{1}{\left| \Omega \right| }\int _\Omega \left| u(x,y,t)-{\bar{u}}(t)\right| \, dx dy, \end{aligned}$$

where

22 $$\begin{aligned} {\bar{u}}(t) = \frac{1}{\left| \Omega \right| }\int _\Omega u(x,y,t)\, dx dy . \end{aligned}$$

For other possible heterogeneity measurements see, e.g., Berding (1987), Murray (2003), Krause et al. (2020). Note that conservation of the cell population means ${\bar{u}}(t) =1$, following nondimensionalisation. Values reported for the heterogeneity measurement *M* are those determined at the end of the simulation.

2D simulations were performed for four scenarios, as summarised in Table 1. First, we investigated the impact of coupling chemotaxis with a Turing system for four possible coupling parameter combinations $\epsilon _{1,2}$, and varying chemosensitivity. Second, we considered a case in which the Turing system alone would not exhibit patterns, but where pattern formation can be induced by coupling with chemotaxis. In the third and fourth scenarios the influence of critical activator–inhibitor interactions was investigated. Specifically, we varied the parameter $\epsilon _{3}$ for cases in which upregulation of the inhibitor is present or not.

**Table 1:** Four different parameter scenarios considered in the 2D simulations

Scenario	*a*	*c*	$\epsilon _{1}$	$\epsilon _{2}$	$\epsilon _{3}$	$D_{w}$	$\chi$
1	0.2	1.3	{0,1}	{0,1}	1	40	0–50
2	1.0	0.5	{0,1}	{0,1}	1	1	0–50
3	0.2	0.2	1	1	0.05–1	1	{2,5,20,50}
4	0.2	0.2	1	0	0.05–1	1	{2,5,20,50}

The remaining parameters are fixed as $\gamma = 2200$, $\beta =4$, and $D_{u}=1$ unless stated otherwise. Set notation indicates a specific set of chosen values, while interval notation indicates a range of values explored or a costant value picked within this range

### Confirmation of Analytical Results and Insights from Simulations

#### 2D Simulations Indicate that Chemotaxis can Speed Up Pattern Formation, but at a Cost of Reduced Pattern Regularity and Temporal Stability

**Fig. 5:**
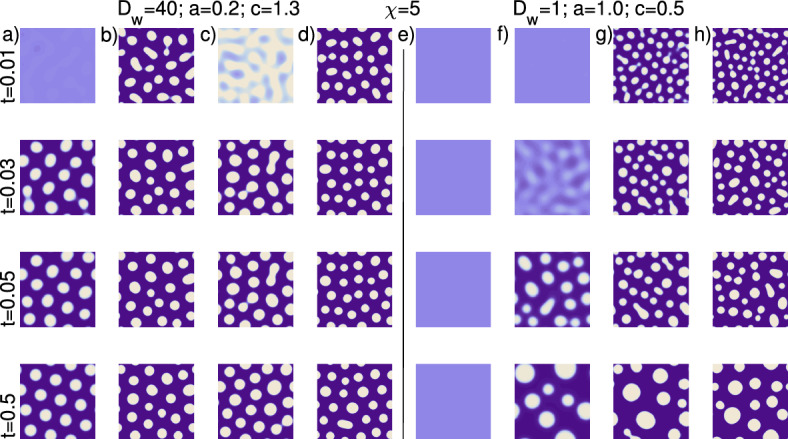
Some results for the parameter choices of Scenarios 1 and 2 from Table 1, with the exception of using a fixed chemosensitivity $\chi =5$. We show the cell density evolution (from $t=0.01$ to $t=0.5$) in a 2D domain for different choices of coupling parameters: $\epsilon _{1,2}=(0,0), \, (0,1),\, (1,0), \,(1,1)$. **a**–**d** Scenario 1: $D_{w}=40$, $a=0.2$, and $c=1.3$. The coupling parameters are $\epsilon _{1}=0$ and $\epsilon _{2}=0$ (**a**), $\epsilon _{1}=0$ and $\epsilon _{2}=1$ (**b**), $\epsilon _{1}=1$ and $\epsilon _{2}=0$ (**c**), $\epsilon _{1}=1$ and $\epsilon _{2}=1$ (**d**). **e**–**h** Scenario 2. Here $D_{w}=1$, $a=1.0$, and $c=0.5$, and the coupling follows the same order as before (Color figure online)

Simulations from Scenario 1 (Table 1) for $\epsilon _{1,2}=0$ (Fig. 5a), show patterns forming around $t=0.05$, resolving into a regular pattern. Introducing a coupling, by either upregulation of the activator or upregulation of the inhibitor, or both, accelerates pattern formation, with clear patterns at $t \lesssim 0.03$ (Fig. 5b–d). Nevertheless, this acceleration comes at an apparent cost of lower pattern regularity: aggregates at the end of the simulation are less evenly spaced, with different sizes and occasional fusions. In Fig. 5e–h we consider parameters from Scenario 2, i.e. outside the Turing space. As expected, pattern formation is not possible in the absence of coupling ($\epsilon _{1,2}=0$, Fig. 5e). Coupling the systems, either by setting $\epsilon _{1}$ or $\epsilon _{2}$ or both to a non-zero value, allows for pattern formation (Fig. 5f–h). Note the relatively late pattern formation in (Fig. 5f), when upregulation via the inhibitor only is given. Notably, the patterns formed are much less regular than those generated within the Turing space, which is characteristic for chemotaxis systems (Hillen and Painter 2009).

#### Simulations Confirm the Analytical Finding that Increasing ChemoSensitivity Facilitates Pattern Formation Inside and Outside of Turing Space

**Fig. 6:**
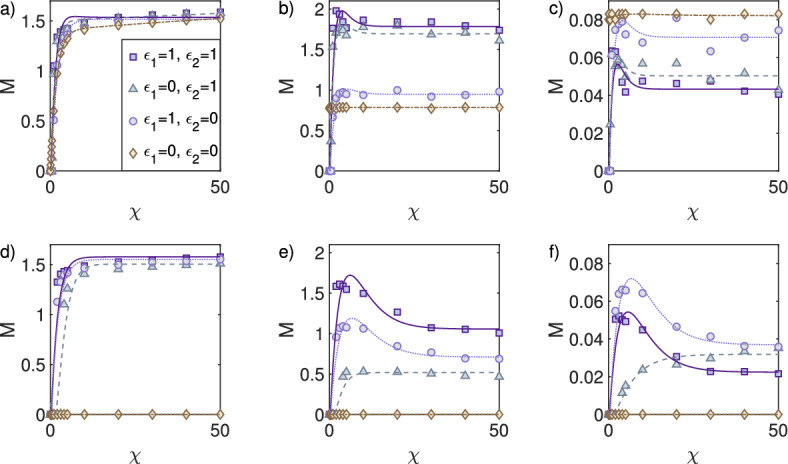
Pattern measurement *M* as a function of chemosensitivity $\chi$. *M* is displayed together with a fitted curve for visualisation purposes ($F(x) = \frac{c_{1}}{c_{2}+ e^{-c_{3}(x-c_{4})}} + \frac{c_{5}}{x^{2}}$) fitted using the Levenberg–Marquardt algorithm Levenberg 1944; Marquardt 1963). The first column shows the results for the cells *u*, the second column the activator *v*, and the third column the inhibitor *w*. **a**–**c** Simulations for Scenario 1 (inside Turing space); $D_{w}=40$, $a=0.2$, and $c=1.3$. **d**–**f** Simulations for Scenario 2 (outside Turing space); $D_{w}=1$, $a=1.0$, and $c=0.5$ (Color figure online)

We consider the heterogeneity measurement for 2D simulations in Scenario 1 and Scenario 2 (Table 1). In both cases we observe that cell density heterogeneity increases with chemosensitivity, eventually reaching a constant value (Fig. 6a, d), likely a result of the boundedness on the cell density due to the presence of volume filling. In Scenario 1, for the activator concentration, taking $\epsilon _{2}=0$ leads to a smaller value of *M* for all $\chi$ when compared to $\epsilon _{2}=1$ (Fig. 6b); this behaviour is reversed for the inhibitor (Fig. 6c). In Scenario 2, for $\epsilon _{1,2}\ne 0$, we observe that *M* is more responsive to the chemosensitivity in the sense that there is a maximum for a given $\chi$ value (Fig. 6e). For $\epsilon _{1,2}=1$ we obtain the largest values of *M* for the activator, and smallest *M* for $\epsilon _{1,2}=0$ (Fig. 6b, e).

#### Simulations Confirm the Analytical Finding that Turing Interaction can Destabilise the Pattern Formation Process

**Fig. 7:**
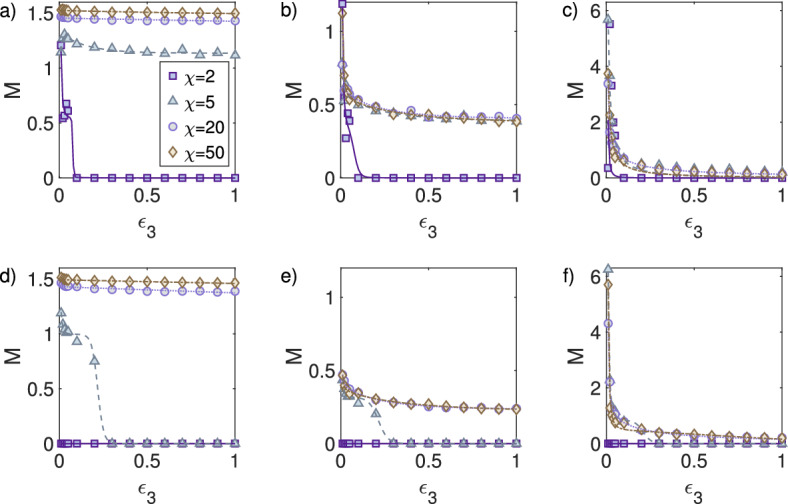
Pattern measurement *M* as a function of coupling the reaction Turing system parameter $\epsilon _{3}$ for different chemosensitivities $\chi$. The first column shows the results for the cells *u*, the second column the activator *v*, and the third the inhibitor *w*. **a**–**c** Simulations for Scenario 3; $D_{w}=1$, $\epsilon _{1}=1$, $\epsilon _{2}=1$, $a=0.2$, and $c=0.2$. **d**–**f** Simulations for Scenario 4; $D_{w}=1$, $\epsilon _{1}=1$, $\epsilon _{2}=0$, $a=0.2$, and $c=0.2$ (Color figure online)

For 2D simulations in Scenario 3 ($\epsilon _{2}=1$) and Scenario 4 with ($\epsilon _{2}=0$), see Table 1, the strength of the reaction between activator and inhibitor ($\epsilon _{3}$) is varied for fixed chemosensitivities $\chi \in \{ 2, \, 5,\, 20,\, 50\}$. In Scenario 3, for small chemosensitivities ($\chi =2$) we observe that for $\epsilon _{3}<0.1$ the heterogeneity measurement *M* is nonzero, but for $\epsilon _{3}>0.1$ it drops to zero. This same behaviour is not observed for larger chemosensitivities $\chi \in \{5,20,50\}$ (Fig. 7a–c). These simulations confirm our analytical findings that the Turing system, for low chemosensitivity, can destabilise the pattern formation process (Fig. 4a–c). Taking the inhibitor secretion to zero $\epsilon _{2}=0$ prevents the system from exhibiting pattern formation for any $\epsilon _{3}$ and small chemosensitivities ($\chi \in \{2,5\}$). Specifically, for $\chi =5$ the heterogeneity measurement *M* drops to zero for $\epsilon _{3}>0.3$. For larger chemosensitivities ($\chi =20$ or $\chi =50$) pattern formation occurs for all values of $\epsilon _{3}$ (Fig. 7d–f).

#### Decreased Cell Motility with Respect to the Chemical System Leads to Stable and Distinct Patterns

We next performed an examination of the effect of the parameters on the time scale of patterning. For easier visualisation of the pattern formation dynamics we reduce to a 1D scenario. All 1D simulations use $\beta =4$ and $\gamma =2200$.

**Fig. 8:**
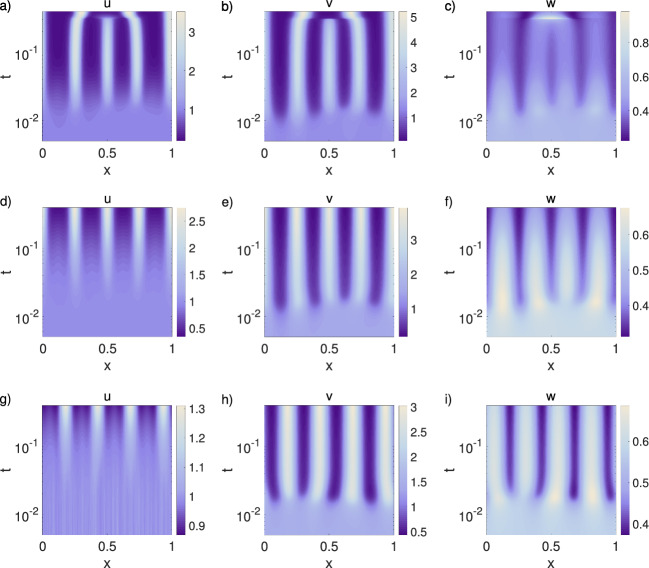
Effect of cell motility being slower than diffusion of chemical components. In the 1D simulations we decreased the diffusion coefficient $D_{u}$ and the chemosensitivity $\chi$ of the cells while the parameters $D_{w}=40$, $a=0.2$, $c=1.3$, and $\epsilon _{1,2,3}=1$ remained fixed. Shown are in each case the cells concentration (*u*), the activator concentration (*v*), and the inhibitor concentration (*w*). **a**–**c** $D_{u}=\chi =0.1$. **d**–**f** $D_{u}=\chi =0.01$. **g**–**i** $D_{u}=\chi =0.001$. Note the different scaling of the colorbar, and that the time axes are displayed in logarithmic scale (Color figure online)

Experiments show that in mice skin patterning the Turing-based patterning develops first, with cells subsequently accumulating underneath activator foci via chemotaxis (Glover et al. 2017). Influential here will be the relative rates of cellular movement to chemical diffusion, for example some studies estimating cell diffusion coefficients to be several orders of magnitude below chemical diffusion coefficients (Chettibi et al. 1994). In this sense, it is relevant to consider the scenario in which the cells are less motile than the reacting chemicals. In order to investigate this, we decrease the cells diffusion coefficient $D_{u}$ and the chemosensitivity $\chi$, choosing for both the values $\{0.1, \, 0.01, \, 0.001\}$. Further, we fixed the inhibitor diffusion coefficient to $D_{w}=40$ and chose $a=0.2$, $c=1.3$, and $\epsilon _{1,2,3}=1$. With this parameter setting the Turing pattern forms first and guides cells to locations of higher activator concentration: chemotaxis is effectively enslaved to the Turing pattern formation, and cell accumulation forms after a molecular prepattern is generated, in line with observations. The Turing process determines completely the location of cell clusters and the resulting patterns are more stable (Fig. 8d–i) than in the case of early and strong chemotaxis (Fig. 8a–c). Notably, though chemotaxis remains strong enough such that the emerging cell clusters become more compact and hence the patterns are more distinct (Figs. 8d–f).

**Fig. 9:**
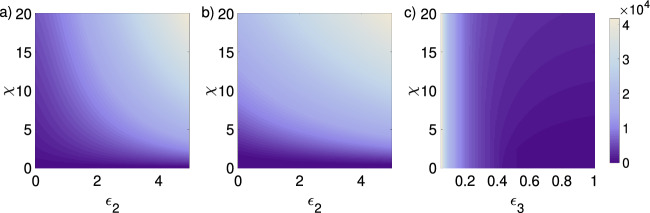
The absolute value of the maximum real part eigenvalue $\max \{{\mathcal {R}}(\lambda )\}$. **a**–**c** The absolute value of the maximum real part eigenvalue obtained using Matlab for varying parameters. **a** Inside Turing space. Impact of $\chi$ and $\epsilon _{2}$ for fixed $D_{w}=40$, $\epsilon _{1,3}=1$, $a=0.2$, and $c=1.3$. **b** Outside Turing space. Impact of $\chi$ and $\epsilon _{2}$ for fixed $D_{w}=1$, $\epsilon _{1,3}=1$, $a=1$, and $c=0.5$. **c** Impact of the reaction term. We varied $\chi$ and $\epsilon _{3}$ but kept the parameters $D_{w}=1$, $\epsilon _{1,2}=1$, $a=0.2$, and $c=0.2$ fixed (Color figure online)

**Fig. 10:**
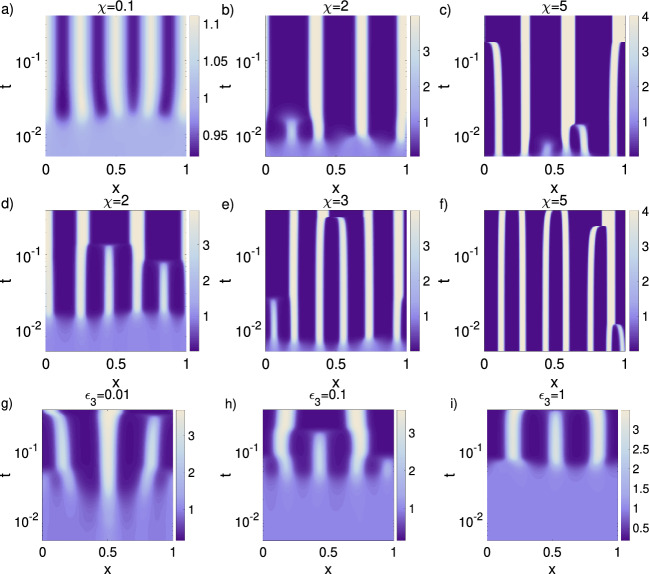
1D simulations for the time scale of pattern formation. **a**–**c** The 1D cell concentration for parameters in Fig. 9a, $\epsilon _{2}=1$, and varying $\chi \in \{0.1,\,2,\,5\}$. **d**–**f** The 1D cell concentration for parameters in Fig. 9b, $\epsilon _{2}=1$ and varying $\chi \in \{2,\,3,\,5\}$. **g**–**i** The 1D cell concentration for parameters in Fig. 9c, $\chi =4$ and varying $\epsilon _{3} \in \{0.01,\,0.1,\,1\}$. Note the different scaling of the colorbar, and that the time axes are displayed in logarithmic scale (Color figure online)

Generally, the magnitude of the largest real part of the eigenvalues $(\max \{{\mathcal {R}}(\lambda )\})$ determines the initial rate of growth of the patterns, and hence provides an indicator of the timescale of patterning (Fig. 9a–c). The eigenvalue plots and simulations are shown for $\gamma =2200$. The 1D simulations provide information on the time scale of patterning for three setups: (i) varying chemosensitivity ($\chi \in \{0.1, \, 2, \, 5 \}$) for parameters inside the Turing space $D_{w}=40$, $a=0.2$, $c=1.3$, and $\epsilon _{1,2,3}=1$ (Fig. 10a–c); (ii) varying chemosensitivity ($\chi \in \{2, \, 3, \, 5 \}$) for parameters outside the Turing space, i.e., $D_{w}=1$, $a=1.0$, $c=0.5$, and $\epsilon _{1,2,3}=1$ (Fig. 10d–f); (iii) a varying reaction term ($\epsilon _{3} \in \{ 0.01, \, 0.1, \, 1\}$) using $D_{w}=1$, $a=c=0.2$, and $\epsilon _{1,2}=1$ for the low chemotaxis regime $\chi =4$ (Fig. 10g–i).

#### All Parameters Influence the Magnitude of the Largest Eigenvalue Inside and Outside of the Turing Space

We observe a dependence on $\epsilon _{2}$ and $\chi$ such that if both values are small we have slow pattern formation, and if both are large we have fast pattern formation (Fig. 9a, b). Considering a small fixed $\epsilon _{2}$ we notice that $\max \{{\mathcal {R}}(\lambda )\}$ increases faster for increasing $\chi$ when the system is outside the Turing space (Fig. 9a) than when it is inside (Fig. 9b). So, as we increase from $\chi = 0$ to $\chi = 20$, for instance, the system outside the Turing space will have a bigger $\max \{{\mathcal {R}}(\lambda )\}$ than the one inside the Turing space for $\epsilon _{2}=1$. On the other hand, we observe an opposite behaviour for a larger secretion of the inhibitor (Fig. 9a, b). Furthermore, the impact of $\epsilon _{3}$ is very strong, and dominates the effect of any change on $\chi$ on the pattern formation dynamics, hereby the patterns form significantly faster for small values of $\epsilon _{3}$, i.e. weak Turing interaction (Fig. 9c).

#### Turing Interaction and Chemotaxis have Opposite Effects on Pattern Formation Dynamics and Long-Term Pattern Stability

The 1D simulations show that inside (Fig. 10a–c) and outside the Turing space (Fig. 10d–f) increasing $\chi$ speeds up pattern formation. Only for very small $\chi$, the Turing process is able to stabilise the pattern in time (Fig. 10a).We also observe that increasing the coupling of the Turing system slows down the patterning process (Fig. 10g–i), and leads to a stable pattern for strong interaction (Fig. 10i). Unstable patterns, i.e., merging spots, are characteristic for chemotaxis systems and are observed when chemotaxis dominates (Fig. 10b–h). In turn, the patterns are stabilised when the Turing process dominates, e.g., for $\chi =0.1$ (Fig. 10a) or $\epsilon _{3}=1$ (Fig. 10i).

*Key observations* Overall, the 1D and 2D simulations confirm the findings from the linear stability analysis: (a) chemotaxis can act as a backup mechanism in case the Turing patterning fails, (b) when chemotaxis is coupled to a Turing system, the patterning is very robust, (c) cellular production of the activator has a stronger impact on pattern formation than production of inhibitor, but inhibitor production is also relevant, (d) increasing the reaction rate $\epsilon _3$ between the chemicals can lead to a stable system without pattern formation when chemosensitivity is low, (e) high chemosensitivity leads to robust pattern formation also for large $\epsilon _3$, (f) decreased cell motility with respect to the chemical system leads to stable and distinct patterns, with the chemotaxis enslaved by the Turing system.

## Discussion

Inspired by recent experimental results, we have considered a coupled reaction–diffusion–chemotaxis system. Stability analysis was carried out for a relatively general formulation, and conditions for pattern formation were obtained. To illustrate these within a specific system, we focussed on a standard set of reaction terms and pattern forming regions were identified for various parameter combinations, including the chemosensitivity, inhibitor diffusion coefficient, and the coupling constants. Patterning could be either enhanced or inhibited through the coupling, indicating a potentially complex set of patterning outcomes when these mechanisms operate in tandem.

A primary motivation lay in the complex cellular and molecular interactions that regulate primary hair follicle patterning in mice. As described in Glover et al. (2017), epidermal and dermal cells interact with a network of growth factors, responsible for the pre-patterning of hair placodes, including bone morphogenic protein (BMP), fibroblast growth factor (FGF), and wingless-related integration site (WNT). Chemotaxis of both dermal and epidermal cells is mediated by widespread transforming growth factor (TGF$\beta$). The model here could be considered to be an abridged description of this system, reduced to the essential coupling between chemotaxis and a reaction–diffusion prepattern mechanism. A natural extension would be to move towards an experimentally-informed model with molecular signalling coupled to multiple distinct cell populations/tissue layers, based on the known interactions (Glover et al. 2017); of course, at that point the dimensionality of the system increases and analysis becomes less tractable. Nevertheless, other models have been developed that consider patterning across separate epithelial and mesenchymal populations, ranging from a simple description as two separate but overlapping variables (e.g. Painter et al. 2018) to more sophisticated description with interfacial transport between the two distinct tissues (Diez et al. 2013).

The proposed model extends mathematical descriptions of pattern formation by including more complex interactions between a spectrum of cellular and molecular populations. This approach follows other trends towards greater elaboration, including: models for feather primordia pattern formation that couple multiple cell populations responding by chemotaxis to interacting molecular regulators (Michon et al. 2008; Painter et al. 2018; Ho et al. 2019; Bailleul et al. 2019); reaction–diffusion models that are structured across tissue layers, accounting for interfacial transport (Diez et al. 2013); coupling of stochastic individual-based model of cell movement to reaction–diffusion models on static and evolving geometries (Macfarlane et al. 2020); reaction–diffusion models extended to multiple ($>2$) molecular regulators (Marcon et al. 2016; Economou et al. 2020; Landge et al. 2020); or, multi-layered and interlinking reaction–diffusion networks (Barrio et al. 1999; Yang and Epstein 2003). Our results reinforce a general notion that greater model sophistication can lead to more flexible patterning: the classic limitations of standard two variable activator–inhibitor systems—such as the requirement for markedly distinct diffusion coefficients and some form of self-catalytic behaviour (Murray 2003)—relax when coupled to a chemotactic population. Strict self-catalysis may no longer be required, and molecular components could have more or less equal diffusion coefficients. On the other hand, we also find that coupling can sometimes allow pattern elimination, e.g. with increased chemotaxis suppressing pattern formation in certain instances. Overall, given that successful morphogenesis of many organs is often contingent on precisely coordinated spatial activity, the interlocking of different patterning mechanisms could allow the exertion of control at distinct stages of development.

A linear stability analysis for the general model using the Routh-Hurwitz conditions and the Descarte’s Rule of Signs (Murray 2003) results in two major pattern conditions. However, when applied to the Schnakenberg secretion model, numerical simulations indicated that within the considered parameter ranges patterning effectively depended only on the simplest condition, which could be described by a second degree polynomial of the squared wave numbers. This allowed us to derive parameter ranges supporting pattern formation analytically using Mathematica software. Simulations were used to validate the analytical findings and to explore the temporal evolution of the patterning process and pattern regularity. By varying the coupling parameters $\epsilon _i$ and the ratio of the diffusion coefficient we studied systems where patterning was dominated by diffusion–driven or chemotaxis-driven instability, or a combination of both. In certain configurations, we found that coupling a chemotaxis process to a reaction–diffusion process could lead to an acceleration in the timescales of pattern development: intuitively, this could occur if the coupling is implemented such that any spatially localised regions of self-activation within the reaction–diffusion system are accompanied by cell accumulations that reinforce this process. Patterning timescales are often somewhat neglected in modelling studies, but embryonic pattern formation of course requires not just the establishment of a spatial pattern, but that it develops within a relevant time scale: visible indications of hair follicles (i.e. gene expression in the epidermis and mesenchymal condensations below) typically appear some 10–20 h from the undifferentiated skin (for example, 18 h in Fig. 1b) and have an interfollicle spacing $\sim 200 \upmu$ m (e.g. see Fig. 1b). For a reaction–diffusion model with plausible values for diffusion coefficients, establishing the spatial pattern within these timescales places critical restrictions on other key rate parameters, such as activator/inhibitor half lives and synthesis rates (see Painter et al. 2012). Since reaction–diffusion pattern formation can be slowed down by various factors, such as gene expression delays (Seirin Lee et al. 2010), any mechanisms for enhancing the timescale of patterning may play an important role to achieve morphogenesis within relevant timescales.

In settings where patterning through the Turing reaction–diffusion system dominates, the pattern is relatively stable as it emerges: elements of the pattern do not significantly shift position, at least during the considered simulation time. Patterning for parameter scenarios where chemotaxis dominates, however, can be characterised by significant movement of cellular foci following their first appearance, including fusion, expansion and extinction events: patterns formed through chemotaxis models are relatively well known for highly dynamic patterning, since strong attraction can occur between neighbouring aggregates (e.g. see Painter 2019). Our findings are consistent with observations from time-lapse microscopy of developing skin. Under normal conditions, where the chemotaxis process is subordinate to a reaction–diffusion prepattern, mouse hair follicle cell aggregates form an apparently fixed and static pattern (Glover et al. 2017). When the pre-patterning mechanism is suppressed, however, the pattern of cell condensates that emerge through chemotaxis in the mesenchyme has a less defined/fused form (Glover et al. 2017). Further, in chicken feather patterning where chemotaxis appears to be a critical part of the pattern-forming process, cellular aggregates are seen to move about and interact after their initial emergence before settling in position (Ho et al. 2019).

Our model does not account for cell proliferation, which can be justified by experimental studies on skin patterning in chicken. Here, proliferation is required to produce a sufficiently high cell density to permit patterning, but in conditions where proliferation is suppressed periodic patterning still occurs in those regions of the tissue where a high enough cell density has been attained (Ho et al. 2019). Also, patterning of hair follicles in mice takes about 10 h, but here cells divide less than once every 24 h (Riddell et al. 2023 and D. Headon, unpublished observation). Proliferation, however, is certainly relevant for the biological system on a larger time scale, and could be included in further work on this system. In the mouse, a space-filling hair development can be crucial for survival. Therefore, characterising the spatial arrangement of the follicles under various chemotaxis effects is relevant for a better understanding of embryo development and should be investigated further in future studies. The model can be further extended by a more detailed description of the growth factor interaction network consisting of approximately 20 compounds (Glover et al. 2017), by including mechanical interaction between epithelial and dermal cells, stochasticity, or three-dimensional effects, like shape or curvature, of the real skin system.

## Data Availability

The datasets generated during and/or analysed during the current study are available from the corresponding author on reasonable request.

## Appendix A: Classical Systems

To provide context to our findings on the full system, we briefly comment on conditions for patterning in the two classical sub-models on which Eq. 1 is constructed. First, consider a two-species reaction–diffusion system of activator(*v*)–inhibitor(*w*) type,

23 $$\begin{aligned} \begin{array}{rcl} \displaystyle \frac{\partial v}{\partial t} &{}= &{} D_v \,\nabla ^{2} v \,+ \, f(v,w)\\ \displaystyle \frac{\partial w}{\partial t} &{}= &{} D_w \,\nabla ^{2} w \,+ \, g(v,w). \end{array} \end{aligned}$$

and denote by $(v^*,w^*)$ a (positive) homogeneous steady state, i.e. $f(v^*,w^*)=g(v^*,w^*)=0$. Standard diffusion–driven (or Turing) instability analysis involves (see, for example, Murray 2003): (i) deriving the linearised equations following a perturbation about the homogeneous steady state, ${\bar{V}} = ({\bar{v}},{\bar{w}}) = (v(x,t)-v^*,w(x,t)-w^*)$; (ii) looking for solutions to the linearised system of the form ${\bar{V}} \sim e^{\lambda t} e^{ikx}$, where the wavenumber *k* relates to the wavelength of patterns, to determine a stability matrix for the eigenvalues $\lambda$; (iii) exploring the conditions under which the steady state is stable following a homogenous perturbation, but unstable following an inhomogenous perturbation. The latter step is equivalent to determining the conditions under which perturbations grow or decay over time, i.e. whether there exist any eigenvalues with positive real components for any valid wavenumber. The resulting set of conditions forms what we refer to here as the Turing space, defined in the following.

### Definition 1

(*Turing space*) The Turing space is defined as the parameter region in which the following inequalities are satisfied (Murray 2003):

24 $$\begin{aligned} f_{v}+g_{w}< & {} 0 \end{aligned}$$

25 $$\begin{aligned} f_{v}g_{w}-f_{w}g_{v}> & {} 0 \end{aligned}$$

26 $$\begin{aligned} D_{w}f_{v}+D_{v} g_{w}> & {} 0 \end{aligned}$$

27 $$\begin{aligned} (D_{w}f_{v}+D_{v}g_{w})^{2}-4D_{v}D_{w}(f_{v}g_{w}-f_{w}g_{v})> & {} 0 \end{aligned}$$

Note that subscripts on the kinetic functions denote partial derivatives evaluated at the steady state, i.e. $f_{v} = \frac{\partial f}{\partial v} \left. \right| _{(v^*,w^*)}$. The term diffusion–driven highlights that adding diffusion induces the instability. Investigating the above conditions leads to general principles for patterning, within the context of a two variable systems. First, the “activator” must have an autocatalytic property, i.e. $f_v > 0$. Second, activator and inhibitor require distinct diffusion ranges, specifically $D_v < D_w$. Third, the sign structure for the signalling interactions must fall into one of two general classes:

28 $$\begin{aligned} \left( \begin{array}{cc} f_v &{} f_w \\ g_v &{} g_w \end{array}\right) \equiv \left( \begin{array}{cc} + &{} - \\ + &{} - \end{array}\right) \quad \text{ or } \quad \left( \begin{array}{cc} + &{} + \\ - &{} - \end{array}\right) , \end{aligned}$$

where the first form is referred to as a pure activator–inhibitor system, and the second form a cross activator–inhibitor system.

An alternative route to patterning is through a chemotaxis-driven autoaggregation (Keller and Segel 1970). Here a minimal system can be constructed from the chemotactic cell population and the associated signalling component, i.e.

29 $$\begin{aligned} {\left\{ \begin{array}{ll} \displaystyle \frac{\partial u}{\partial t} \,&{}=\, D_{u} \nabla ^{2} u \,-\, \chi \cdot \nabla ( u \nabla v)\\ \displaystyle \frac{\partial v}{\partial t} \,&{}=\, D_v \nabla ^{2} v \,+\, \, f(u,v) \end{array}\right. } \end{aligned}$$

For the above, the homogeneous positive steady state is $(u^*,v^*)$, where conservation of mass determines $u^*$ from the initial conditions, i.e. $u^*$ is the mean value of *u*(*x*, 0), when averaged over space. $v^*$ then solves $f(u^*,v^*)=0$. Performing the equivalent stability analysis leads to a set of conditions under which chemotaxis can drive self-organisation, defined here as the chemotaxis space.

### Definition 2

(*Chemotaxis space*) The chemotaxis space is defined as the parameter region in which the following inequalities are satisfied:

30 $$\begin{aligned} f_{v}< & {} 0 \end{aligned}$$

31 $$\begin{aligned} \chi f_u u^* + f_v D_u> & {} 0 \end{aligned}$$

The classical autoaggregation scenario involves a cell population that produces its own attractant (although it could also include a cell population that downregulates its own repellent). The above conditions then stipulate a threshold on the chemotactic sensitivity, along with the density of the chemotactic population and the rate at which attractant is produced. Note that there is a distinction on the sign of $f_v$ in the two spaces above: for the Turing space, the need for an autocatalysis demands $f_v >0$, while in the chemotaxis space we require $f_v<0$ for stability of the steady state under homogeneous perturbations.

As a point of remark, we note that the chemotaxis system without cellular growth terms will yield a zero eigenvalue for the linear stability analysis that follows a homogeneous perturbation to the steady state. As such, the steady state is not asymptotically stable and certain perturbations (i.e. those that lead to a change in the cell mass) will result in a shift in the position of the steady state. For these reasons we have imposed initial conditions such that the perturbation to the steady state has zero mean and, consequently, the steady state remains at a fixed position. A more formal analysis, for example using centre manifold theory is beyond the aims of the current work. The same reasoning applies for the coupled chemotaxis-reaction–diffusion system studied in Sect. 3.1.

## Appendix B: Dimensional Analysis

We derive the Turing Schnakenberg system and couple it to the chemotaxis model. Subsequently, by nondimensionalisation we obtain (Eq. 3).

The Schnakenberg system is a hypothetical chemical reaction (Schnakenberg 1979) for given general chemicals X, A, B, and Y (Eq. 32), that can exhibit limit-cycle behaviour (periodic time oscillations), and is used as a prototype reaction for Turing-based patterning (Murray 2003). Reactions are assumed to evolve as

32

It is assumed that the concentrations $[A]=A$ and $[B]=B$ are approximately constant, i.e., the chemical reactions are performed in an environment with an abundance of A and B. Applying the Law of Mass Action to these reactions and assuming diffusion, we can model the dynamics through a system of partial differential equations for varying concentrations $[X]=V(x,t)$ and $[Y]=W(x,t)$, in space *x* and time *t*, for diffusion coefficients $D_{v}$ and $D_{w}$ (Eq. 33).

33 $$\begin{aligned} {\left\{ \begin{array}{ll} \dfrac{\partial V}{\partial t} \,&{}=\, D_{v} \nabla ^{2}V \,+\, k_{2}A \,-\, k_{1}V \,+\, k_{4}V^{2}W \\ \dfrac{\partial W}{\partial t} \,&{}=\, D_{w} \nabla ^{2}W \,+\, k_{3}B \,-\, k_{4}V^{2}W \end{array}\right. } \end{aligned}$$

We assume that the cells $U=U(x,t)$ follow the *V* gradient, with chemosensitivity $\chi$, spread with diffusion coefficient $D_{u}$, and that they secrete both chemicals *V* and *W* with rates $r_{1}$ and $r_{2}$, respectively. Furthermore, the cell density is bounded using a volume-filling formulation (Painter and Hillen 2002) with constant $\beta$. The resulting coupled chemotaxis and Turing systems is represented in Eq. 34.

34 $$\begin{aligned} {\left\{ \begin{array}{ll} \dfrac{\partial U}{\partial t} \,&{}=\, D_{u} \nabla ^{2}U \,-\, \chi {\varvec{\nabla }} \,\cdot \,((1-U/\beta )U\, {\varvec{\nabla }}V) \\ \dfrac{\partial V}{\partial t} \,&{}=\, D_{v} \nabla ^{2}V \,+\, r_{1}U\,+\, k_{2}A \,-\, k_{1}V \,+\, k_{4}V^{2}W \\ \dfrac{\partial W}{\partial t} \,&{}=\, D_{w} \nabla ^{2}W \,+\, r_{2}U\,+\, k_{3}B \,-\, k_{4}V^{2}W \end{array}\right. } \end{aligned}$$

Consider the dimensional system presented in Eq. 34 and the parameters units in terms of *M* as mass unit, *L* as space unit, and *T* as time unit (Table 2).

**Table 2:** Dimensional analysis of parameters in terms of *M* as mass unit, *L* as space unit, and *T* as time unit

Variable/parameter	Dimension
*x*	*L*
*t*	*T*
$U,\, V,\, W$	$M/L^{3}$
$D_{u},\, D_{v},\, D_{w}$	$L^{2}/T$
$\chi$	$L^{5}/(TM)$
$\beta$	$M/L^{3}$
$r_{1},\, r_{2},\, k_{1},\, k_{2}, \,k_{3}$	1/*T*
$A, \,B$	$M/L^{3}$
$k_{4}$	$L^{6}/(TM^{2})$

We introduce the following dimensionless variables:

$$\begin{aligned} x^{*}=\frac{x}{L} , \quad t^{*}=\frac{D_{v}t}{L^{2}}, \quad v=V\sqrt{\frac{k_{4}}{k_{1}}}, \quad w=W\sqrt{\frac{k_{4}}{k_{1}}}, \quad u=\frac{U}{u^*} \end{aligned}$$

In the above, $u^*$ is the constant initial distribution of the cells. Substituting into the original system Eq. 34 we obtain the dimensionless system in Eq. 35.

35 $$\begin{aligned} {\left\{ \begin{array}{ll} \dfrac{\partial u}{\partial t^{*}} = \dfrac{D_{u}}{D_{v}}\, \nabla ^{2}u - \Big ( \dfrac{\chi }{D_{v}} \sqrt{\dfrac{k_{1}}{k_{4}}}\Big ) \, {\varvec{\nabla }} \,\cdot \,\Big [ u \Big ( 1- \dfrac{u}{\beta / u^*} \Big ) \, {\varvec{\nabla }}v \Big ] \\ \dfrac{\partial v}{\partial t^{*}} = \nabla ^{2}v + \Bigg ( \dfrac{L^{2}k_{1}}{D_{v}}\Bigg ) \Bigg [ \dfrac{A k_{2}}{k_{1}}\sqrt{\dfrac{k_{4}}{k_{1}}} \Big (1 + \dfrac{r_{1}u^*}{A k_{2}}u \Big ) - v + v^{2}w \Bigg ]\\ \dfrac{\partial w}{\partial t^{*}} = \dfrac{D_{w}}{D_{v}}\, \nabla ^{2}w + \Bigg ( \dfrac{L^{2}k_{1}}{D_{v}}\Bigg ) \Bigg [ \dfrac{B k_{3}}{k_{1}}\sqrt{\dfrac{k_{4}}{k_{1}}} \Big (1 + \dfrac{r_{2}u^*}{B k_{3}}u \Big ) - v^{2}w \Bigg ] \end{array}\right. } \end{aligned}$$

We consider the new parameters:

$$\begin{aligned} D^{*}_{u}&=\dfrac{D_{u}}{D_{v}}, \quad D^{*}_{w}=\dfrac{D_{w}}{D_{v}}, \quad \gamma = \dfrac{L^{2}k_{1}}{D_{v}}, \quad \chi ^{*} = \dfrac{\chi }{D_{v}}\sqrt{\dfrac{k_{1}}{k_{4}}}, \quad \beta ^{*} = \dfrac{\beta }{u^*}\\ a&=\dfrac{A k_{2}}{k_{1}}\sqrt{\dfrac{k_{4}}{k_{1}}} ,\quad \epsilon _{1}= \dfrac{r_{1}u^*}{A k_{2}}, \quad c= \dfrac{B k_{3}}{k_{1}}\sqrt{\dfrac{k_{4}}{k_{1}}} , \quad \epsilon _{2}= \dfrac{r_{2}u^*}{B k_{3} \ ,} \end{aligned}$$

And insert into Eq. 35 to obtain the dimensionless system in Eq. 36. We drop the $*$’s for notational simplicity. We also add a nondimensional parameter $\epsilon _{3}$ that controls the autocatalytic reaction between *v* and *w*.

36 $$\begin{aligned} {\left\{ \begin{array}{ll} \dfrac{\partial u}{\partial t} = D_{u} \nabla ^{2}u - \chi {\varvec{\nabla }} \,\cdot \,(u(1-u/\beta )\, {\varvec{\nabla }}v)\\ \dfrac{\partial v}{\partial t} = \nabla ^{2}v + \gamma (a (1+\epsilon _{1} u)-v + \epsilon _{3} v^{2}w)\\ \dfrac{\partial w}{\partial t} = D_{w} \nabla ^{2}w + \gamma (c(1+\epsilon _{2}u) - \epsilon _{3} v^{2}w) \end{array}\right. } \end{aligned}$$

## Appendix C: Detailed Linear Stability Analysis of the Full Model

### The Condition C<0

The coefficients *A*, *B*, and *C* of the characteristic polynomial of the stability matrix Eq. 6 can be computed as

$$\begin{aligned} A&= k^{2} (D_{v}+D_{u}+D_{v}) - (f_{v}+g_{w})\, ,\\ B&= k^{4}(D_{v} D_{w} + D_{u} (D_{v} + D_{w})) - k^{2} [\chi u^* f_{u} + D_{w}f_{v}\\&\quad + D_{u}(f_{v}+g_{w})+D_{v} g_{w}] +(-f_{w}g_{v}+f_{v}g_{w}) \\ C&= D_{u}D_{v}D_{w} k^{6} - k^{4}[\chi u^* D_{w}f_{u} + D_{u}(D_{w}f_{v}+D_{v}g_{w})] \\&\quad + k^{2}[\chi u^*(-f_{w}g_{u}+f_{u}g_{w}) + D_{u}(-f_{w}g_{v}+f_{v}g_{w})] \, . \end{aligned}$$

Observe that $A>0$ for all *k*, due to positivity of the diffusion coefficients along with the condition Eq. 24. We employ the Routh-Hurwitz condition (Eq. 37) to study the roots of the polynomial given in Eq. 7.

37 $$\begin{aligned} \max \{{\mathcal {R}}(\lambda )\} <0 \quad \Leftrightarrow \quad A>0; \, C>0; \, AB-C>0 \ . \end{aligned}$$

Given that we require at least one eigenvalue with real positive part, one of the Routh-Hurwitz conditions should be broken, that is, $C<0$ or $AB-C<0$.

First we consider the easier case $C<0$. For this, we note first that *C* is a polynomial in $k^2$ and can be written as $C(k^{2}) = k^{2}(ak^{4}+bk^{2}+c) <0$. We study the polynomial Eq. 38 in $q=k^{2}$ with the coefficients given by Eqs. 39–41, e.g.,

38 $$\begin{aligned} {\bar{C}}(q)&\,=\,a_{1}q^{2}+b_{1}q+c_{1} \ , \text{ with } \end{aligned}$$

39 $$\begin{aligned} a_{1}&\,=\,D_{u}D_{v}D_{w}\, \end{aligned}$$

40 $$\begin{aligned} b_{1}&\,=\,-[\chi u^* D_{w} f_{u} + D_{u} (D_{w} f_{v} + D_{v}g_{w})]\, \end{aligned}$$

41 $$\begin{aligned} c_{1}&\,=\,[\chi u^*(-f_{w}g_{u}+f_{u}g_{w}) + D_{u}(f_{v}g_{w}-f_{w}g_{v})] \ . \end{aligned}$$

The roots of the second degree polynomial Eq. 38 can be explicitly calculated:

42 $$\begin{aligned} q_{1}\,=\, \frac{-b_{1} + \sqrt{\Delta _{1}}}{2a_{1}} \, , \quad q_{2}\,=\, \frac{-b_{1} - \sqrt{\Delta _{1}}}{2a_{1}}\, , \quad \quad \text {where } \Delta _{1} = b_{1}^{2}-4a_{1}c_{1}\,. \end{aligned}$$

Observe that $a_{1}>0$. Accordingly, the polynomial Eq. 38 is a parabola with a global minimum. For there to be at least one positive root, a necessary condition is to have at least one change in the coefficients’ sign, that is, either $b_{1}<0$ or $c_{1}<0$. Further, the minimum must be negative, $C_{min}<0$, which will occur for $\Delta _{1}>0$ in Eq. 42. Hence ${\bar{C}}(q)<0$ for at least one *q*, if its coefficients satisfy Eq. 43:

43 $$\begin{aligned} (b_{1}<0 \quad \text {or} \quad c_{1}<0) \quad \text {and} \quad \Delta _{1} = b_{1}^{2}-4a_{1}c_{1}>0 \ . \end{aligned}$$

Thus, the case $C<0$ can generate two possible routes to pattern formation. First, consider the case $c_1 <0$. Here (using $a_1 >0$) the condition $\Delta _{1}>0$ is automatically satisfied and we therefore reduce to the sufficient (but not necessary) condition for pattern formation given by Eq. 8. Second, we consider a route to instability through $b_1 <0$. Here, $\Delta _1>0$ is not automatically satisfied and an instability will require the conditions given by Eqs. 9 and 10.

### The Condition AB-C<0

Next we consider the possibility for $AB-C<0$. Our approach here is very similar to the one in Othmer and Scriven (1969). Denoting $q=k^2$, we write $AB-C$ as a third degree polynomial *R*(*q*),

44 $$\begin{aligned} R(q)&=a_{2}q^{3}+b_{2}q^{2}+c_{2}q+d_{2} \end{aligned}$$

45 $$\begin{aligned} a_{2}\,&=\,-D_{u} D_{v} D_{w} + (D_{u} + D_{v} + D_{w}) (D_{v} D_{w} + D_{u} (D_{v} + D_{w})) \end{aligned}$$

46 $$\begin{aligned} b_{2}\,&=\,[\chi D_{w} f_{u} - (D_{v} D_{w} + D_{u} (D_{v} + D_{w})) (f_{v} + g_{w}) + D_{u} (D_{w} f_{v} + D_{v} g_{w})\nonumber \\&\quad + (D_{u} + D_{v} - D_{w}) (\chi f_{u} + D_{w} f_{v} - D_{u} (f_{v} + g_{w}) - D_{v} g_{w})] \end{aligned}$$

47 $$\begin{aligned} c_{2}\,&=\,[(f_{v}+g_{w})(\chi f_{u} + D_{u}f_{v} +D_{w}f_{v}+D_{u}g_{w}+D_{v}g_{w}) \\ \nonumber&\quad +\, \chi (f_{w}g_{u}-f_{u}g_{w})+ (D_{v}+D_{w})(-f_{w}g_{v}+f_{v}g_{w})] \end{aligned}$$

48 $$\begin{aligned} d_{2}\,&=\, -(f_{v} + g_{w}) (f_{v} g_{w} - f_{w} g_{v}) \end{aligned}$$

Equation 11 has turning points at ${\bar{q}}_{1}$ and ${\bar{q}}_{2}$, and an inflection point at ${\bar{q}}_{3}$:

49 $$\begin{aligned} {\bar{q}}_{1,2} = \frac{-b_{2}\pm \sqrt{\Delta _{2}}}{3a_{2}}\, , \quad {\bar{q}}_{3}=\,-\,\frac{b_2}{3a_{2}}\, , \quad \text {where}\quad \Delta _{2}=b_{2}^{2}-3a_{2}c_{2} \ . \end{aligned}$$

We note that the coefficients $a_{2}$ and $d_{2}$ are always positive. The coefficient $d_{2}$ dictates where the curve intersects with the vertical axis, i.e. a positive point. Next, we apply Descartes’ Rule of Signs for the other coefficients $b_{2}$ and $c_{2}$, see Table 3. If $b_{2}$ and $c_{2}$ are always positive, then $AB-C<0$ is not possible. Noting Table 3, for $b_{2}<0$ or $c_{2}<0$, when $\Delta _{2}$ in Eq. 49 is negative, *R*(*q*) increases monotonically with *q* and the single real root is negative. Hence, for positive *q* the curve *R*(*q*) does not attain any negative value. When $\Delta _{2}>0$ and $b_{2}<0$, both turning points are positive, but we also need the requirement $R({\bar{q}}_{1})<0$ to lie in the pattern region. The case in which $\Delta _{2}>0$ and $b_{2}>0$ is more restricted due to there being only one positive turning point, so besides $c_{2}<0$ we also need $R({\bar{q}}_{3})<0$ in this case.

**Table 3:** Descartes Rule of Signs applied to the second degree polynomial *R*(*q*)

Case	$a_{2}$	$b_{2}$	$c_{2}$	$d_{2}$	Real positive	Real negative	Complex
1	+	+	+	+	0	3/1	0/2
2	+	+	–	+	2/0	1	0/2
3	+	–	+	+	2/0	1	0/2
4	+	–	–	+	2/0	1	0/2

## Appendix D: Numerical Methods

The PDE system at hand in this study is of taxis-diffusion–reaction type. Numerical methods for systems of this class are developed, analysed as well as tested and compared with other schemes in applications to various biomedical models in Gerisch (2001), Gerisch and Chaplain (2006). Based on these works, the numerical method has been used in numerous biomedical projects and applications by ourselves and other authors, and the corresponding software provides efficient, accurate, and reliable simulation results, see, for instance, Peiffer et al. (2011), Painter et al. (2015), Domschke et al. (2017), Villa et al. (2022). This software, written as a combination of Matlab and Fortran code, is employed for the simulations in this work here. It is available from the authors upon request.

Characteristics of the PDE system at hand highlight the requirements on any numerical method for the numerical approximation of solutions of taxis-diffusion–reaction systems. Firstly, the PDE system is mass-conservative and that should be respected by the numerical method. Secondly, the system is highly nonlinear in its chemotactic transport and reaction terms. Furthermore, while being of parabolic type due to the positive diffusion coefficients, the system can become chemotaxis-dominated (typically referred to as advection-dominated) and steep moving fronts may evolve. The resolution of these fronts is a computational challenge and our scheme in particular avoids non-physical oscillations in the numerical solution and also preserves the non-negativity of the initial conditions in the numerical solution over time. The latter property holds for the solution of our PDE problem and is a natural demand on any mathematical model describing the spatio-temporal evolution of densities and/or concentrations.

The numerical approach follows the method of lines (MOL) methodology, where we, firstly, discretise the PDE system in space on a spatial grid and, secondly, integrate the resulting ordinary differential equation (ODE) system—the so-called MOL-ODE—in time. Thus we finally arrive at a fully discrete (in space and time) numerical solution. A spatially uniform grid is used by the scheme and kept fixed in time. For spatially one-dimensional simulations the unit interval is subdivided into *N* intervals (grid cells) of equal length, while for spatially two-dimensional simulations the unit square is subdivided into $N\times N$ squares of equal size. Since the PDE system here has three equations, the MOL-ODE thus has dimension 3*N* and $3N^2$ in the spatially one- and two-dimensional setting, respectively. In particular in two spatial dimensions this quickly gives rise to a very high-dimensional ODE system and the time-integration scheme must be capable to cope with that, see below.

The PDE system, written in divergence form, is discretised in space on the given spatial grid using a Finite Volume scheme. The discretised system (MOL-ODE) in this case describes the evolution of the grid cell averages of the three PDE components. Changes to the average mass in each grid cell arise either through reaction terms, and we use the grid cell average to evaluate these terms, or through fluxes (diffusion and chemotaxis) across the grid cell interfaces. This construction ensures that the discretization respects the mass-conservation of the underlying PDE. The diffusive flux accross a grid cell interface is approximated using a central difference of the grid cell averages in the adjacent grid cells. This amounts to a standard second-order central difference approximation of the diffusion terms. For the computation of the chemotaxis fluxes we use a nonlinear discretisation, an upwind-biased discretisation with van Leer limiter function, to ensure that the resulting MOL-ODE is positive and exhibits no non-physical oscillations in its solution. This is approach is based on Hundsdorfer et al. (1995), Sweby (1984) and described and discussed, including the incorporation of boundary conditions, in detail in Gerisch and Chaplain (2006).

The initial conditions for the MOL-ODE are taken as grid cell centre values of the initial condition of the PDE system. This completes the definition of the initial value problem (IVP) for the MOL-ODE which represents, away from local extrema of the solution, a second-order approximation of the PDE system and thus achieves a high accuracy while maintaining positivity of the solution. The IVP for the MOL-ODE resulting from the above spatial discretisation is a stiff system, in particular due to the diffusion term but potentially also from stiff reaction terms. It is also strongly nonlinear due to the reaction terms and due to the taxis term and its discretisation. As a stiff system, the IVP should be numerically integrated in time using an implicit scheme because explicit schemes would require excessively small time steps to be taken for a successful (i.e. stable) and sufficiently accurate numerical solution and lead to unreasonably long computing times.

Here, as in many earlier studies involing taxis-diffusion–reaction systems, we use the time integration scheme ROWMAP, see Weiner et al. (1997). ROWMAP is a linearly-implicit Runge–Kutta method of order four with automatic time-step size control in order to efficiently satisfy a user defined tolerance threshold. Furthermore, ROWMAP is a matrix-free method as the iterative solution of the (linear) equation systems only requires approximated Jacobian matrix (of the ODE’s right-hand side) times vector products. These products are computed efficiently by ROWMAP itself. Thus the user only needs to provide an implementation of the right-hand side of the ODE system and in particluar neither the Jacobian itself nor a Jacobian sparsity pattern are required. All these feature taken together make ROWMAP a very user-friendly, robust, and efficient IVP solver.

As a final point, we report on the values of some parameters of the numerical scheme used in this study. The number of grid cells per unit length was $N=250$. The ROWMAP tolerance threshold was set to $10^{-6}$ and its initial time step size to $10^{-4}$ for one-dimensional and $10^{-2}$ for two-dimensional simulations. Finally, the simulations were run to a final time of $T=0.4$ for one-dimensional and $T=0.5$ for two-dimensional simulations.

## Appendix E: Code Access

The plotted data, the code used for visualization, as well as Mathematica scripts used to explore the regions for pattern formation can be accessed from the GitHub repository under the link: https://github.com/mileknz/RD_chemotaxis. These Mathematica scripts also contain the patterning conditions for the Schnakenberg system. The GitHub repository also includes a file listing the full parameters set for each figure. The code for the numerical treatment and simulation of the PDE systems is available for research purposes upon request.
